# Mapping suitability for Buruli ulcer at fine spatial scales across Africa: A modelling study

**DOI:** 10.1371/journal.pntd.0009157

**Published:** 2021-03-03

**Authors:** Hope Simpson, Earnest Njih Tabah, Richard O. Phillips, Michael Frimpong, Issaka Maman, Edwin Ampadu, Joseph Timothy, Paul Saunderson, Rachel L. Pullan, Jorge Cano

**Affiliations:** 1 London School of Hygiene and Tropical Medicine, London, United Kingdom; 2 National Yaws, Leishmaniasis, Leprosy and Buruli ulcer Control Programme, Cameroon; 3 School of Medical Sciences, Kwame Nkrumah University of Science and Technology, Kumasi, Ghana; 4 National Reference Laboratory for Buruli Ulcer Disease in Togo, Ecole Supérieure des Techniques Biologiques et Alimentaires (ESTBA), Laboratoire des Sciences Biologiques et des Substances Bioactives, Université de Lomé, Lomé, Togo; 5 National Buruli Ulcer Control Program, Ghana Health Service, Accra, Ghana; 6 Accelerating Integrated Management (AIM) Initiative, Accra, Ghana; Swiss Tropical and Public Health Institute, SWITZERLAND

## Abstract

Buruli ulcer (BU) is a disabling and stigmatising neglected tropical disease (NTD). Its distribution and burden are unknown because of underdiagnosis and underreporting. It is caused by *Mycobacterium ulcerans*, an environmental pathogen whose environmental niche and transmission routes are not fully understood. The main control strategy is active surveillance to promote early treatment and thus limit morbidity, but these activities are mostly restricted to well-known endemic areas. A better understanding of environmental suitability for the bacterium and disease could inform targeted surveillance, and advance understanding of the ecology and burden of BU. We used previously compiled point-level datasets of BU and *M*. *ulcerans* occurrence, evidence for BU occurrence within national and sub-national areas, and a suite of relevant environmental covariates in a distribution modelling framework. We fitted relationships between BU and *M*. *ulcerans* occurrence and environmental predictors by applying regression and machine learning based algorithms, combined in an ensemble model to characterise the optimal ecological niche for the disease and bacterium across Africa at a resolution of 5km x 5km. Proximity to waterbodies was the strongest predictor of suitability for BU, followed potential evapotranspiration. The strongest predictors of suitability for *M*. *ulcerans* were deforestation and potential evapotranspiration. We identified patchy foci of suitability throughout West and Central Africa, including areas with no previous evidence of the disease. Predicted suitability for *M*. *ulcerans* was wider but overlapping with that of BU. The estimated population living in areas predicted suitable for the bacterium and disease was 46.1 million.

These maps could be used to inform burden estimations and case searches which would generate a more complete understanding of the spatial distribution of BU in Africa, and may guide control programmes to identify cases beyond the well-known endemic areas.

## Author summary

Like many neglected tropical diseases primarily affecting the rural poor, Buruli ulcer (BU) is under-detected and under-reported within routine health information systems. As such, the burden and distribution are not fully known, impeding appropriate targeting of health resources, control, and care for people affected. Having previously evaluated and mapped the existing evidence for BU and its causative agent *M*. *ulcerans*, we concluded that the disease was likely to occur beyond the range of known endemic areas. However, we were left with the question of where exactly these undetected cases might be occurring. Answering this question required a more fine-scale approach: BU is highly focal, presumably due to local variation in the environmental factors which determine suitability for *M*. *ulcerans* survival and transmission to humans. We used the compiled evidence and geographical datasets to build statistical models representing the relationship between environmental factors and previously reported cases. This allowed us to define the ecological niche of BU, and subsequently to identify areas across Africa where this niche was met, providing suitable conditions for the disease. We constructed separate models of suitability for *M*. *ulcerans*, using locations where its DNA had been detected in environmental sources. Unsurprisingly, suitability for *M*. *ulcerans* was predicted to be wider than, but geographically overlapping with that for BU. This implies that beyond the conditions necessary for survival of the bacterium, additional factors are required for transmission to humans. The high-resolution suitability maps we present are intended to guide case search activities which may identify endemic areas beyond the known endemic range. Data on the true prevalence of BU from targeted case searches within predicted-suitable areas will also allow us to validate and refine the models, and potentially to predict the probability of cases occurring within predicted suitable areas.

## Introduction

Buruli ulcer (BU) is a chronic necrotizing disease of the skin and soft tissue, which causes debilitating symptoms and sequelae, associated with a high burden of morbidity and stigma for patients and economic costs for affected households [1–3]. These impacts are felt particularly strongly in impoverished rural communities with poor access to health services [3,4]. The infectious agent is *Mycobacterium ulcerans*, a slow-growing environmental bacterium which appears to be transmitted from aquatic environments to humans by penetration of the skin, although the exact pathways are not fully understood and are likely to be diverse [1,5,6]. The main control strategy is active case finding in endemic areas to promote early case detection and effective treatment, which limits disease progression [7,8]. BU occurs mostly in tropical and subtropical areas of West and Central Africa, with smaller foci in parts of Asia, South America, the Western Pacific and Australasia [9]. However, the disease is recognised to be underdiagnosed and under-reported, and may occur undetected in other parts of the world [9–12].

In the 1950’s and 60’s, large numbers of cases occurred in Nakasongola District in Uganda, but the incidence of disease in this area then declined and has apparently not resurged since (S1 and S2 Figs) [13]. In West Africa, the highest incidence was reported in the mid 1990’s and appears to have been declining since 2008 [13]. The distribution of BU is presumably linked to environmental suitability- the availability of appropriate conditions- for *M*. *ulcerans* survival and replication, as well as to human and environmental factors favouring transmission [14].

On a continental scale, BU appears to be limited by climatic factors: it is mostly restricted to tropical and subtropical regions and is absent from arid areas [15]. Within endemic areas, the disease shows a highly focal distribution [16–18], but reasons for this are not well understood, since the precise niche and transmission routes of *M*. *ulcerans* have been difficult to characterise [19]. The pathogen has only been cultured from environmental and animal samples a handful of times [20–22], although it has been detected by PCR in aquatic environments of endemic and non-endemic areas, and in a wide range of potential hosts including mammals, fish, amphibians, and aquatic and terrestrial insects [23–27]. Consistent with the ecology of an environmental pathogen, the distribution of *M*. *ulcerans* in the environment appears to be wider than that of BU, suggesting that factors beyond environmental suitability for *M*. *ulcerans* are required for transmission [14,15,28].

Our understanding of the pathways of BU infection is also limited, partly by its long and variable incubation period, which makes it difficult for patients and clinicians to attribute particular events or activities to disease acquisition [29]. Local spatial analysis has identified several environmental variables associated with increased BU incidence, primarily proximity to rivers, as well as environmental disturbance and land-use changes including deforestation, urbanisation, agriculturalization, damming of rivers and mining [30,31]. Case control studies have identified contact with unprotected waterbodies as a risk factor for disease [32], suggesting that activities which bring people into contact with water sources harbouring *M*. *ulcerans* increase the risk of disease acquisition [33–35].

Given the recognised scale of BU under-detection and under-reporting, it is likely that the disease occurs beyond the known range of reported cases. A better understanding of potential suitability for the pathogen in the environment and the disease in humans would help to improve its surveillance and control in countries where is known to be endemic. Furthermore, characterisation of the environmental factors linked to suitability for *M*. *ulcerans* and BU may reveal areas at risk of disease emergence, or harbouring unrecognised cases.

In this investigation, we aim to identify environmental factors which characterise the environmental niche of *M*. *ulcerans* and BU disease in humans, and to model their respective relationships with *M*. *ulcerans* and BU occurrence. These analyses will be used to identify areas of continental Africa which may be suitable for *M*. *ulcerans* or BU based on their environmental characteristics.

## Methods

### Data on Buruli ulcer and *M*. *ulcerans* distribution

We used previously compiled datasets of point locations of recorded occurrences of BU disease in humans, and of detection of *M*. *ulcerans* genetic material in biotic and abiotic environmental samples [9,36]. The datasets were compiled through a systematic review [9] and the BU dataset was supplemented with surveillance data from BU control programmes in Ghana, Nigeria and Cameroon. The literature search was updated in October 2020.

BU occurrence locations were restricted to those where BU infection was confirmed by a positive result for PCR targeting IS2404, or histopathology consistent with BU disease. To explore the model’s sensitivity to the case definition, we repeated the analysis using all locations where clinically diagnosed BU had been reported. We hereon refer to the two datasets as ‘*confirmed occurrences*’ and ‘*all occurrences*’ respectively.

The environmental dataset was restricted to locations where *M*. *ulcerans* DNA had been identified and distinguished from that of other mycobacteria: either by multiplex qPCR assays quantifying the relative copy numbers of IS2404, IS2606 and the KR-B domain [37]; by variable nucleotide tandem repeat (VNTR); or mycobacterial interspersed repetitive unit (MIRU) typing [38,39]. We hereon refer to this dataset as ‘*environmental occurrences*’.

All records were restricted to locations with reliable geographical coordinates and deduplicated by geographical location.

### Environmental datasets used in ecological modelling

We assembled gridded datasets of 14 environmental variables considered relevant to the ecological niche of the bacterium or disease [19]. These included four variables considered to characterise the tropical and subtropical biomes from where the majority of BU cases in Africa have been reported [40]: minimum and maximum temperature [41,42], the aridity index, quantifying atmospheric aridity (the balance of precipitation and atmospheric water demand [43,44]) and potential evapo-transpiration (a measure of atmospheric capacity to remove water from the air through evaporation and transpiration assuming unlimited water availability) [40,43]. Tropical climates are also characterised by the amount of precipitation they experience, so we included indicators of precipitation seasonality and precipitation in the wettest and driest quarters [45], which have been linked to trends in the abundance of *M*. *ulcerans* in the environment and the incidence of BU cases in Cameroon, Ghana, and Uganda [46–48]. We also included indicators of topography which may identify the swampy, stagnant environments where BU is often reported in endemic countries [14,49], specifically elevation [43] and topological wetness index (derived from elevation), representing the potential for each cell to accumulate water based on its elevation relative to surrounding cells and the potential for drainage [50]. Since particular vegetation and landcover types have previously been associated with BU endemicity [31,49], we included the enhanced vegetation index (EVI) which quantifies vegetation cover [51,52]. We calculated Euclidean (straight line) to the nearest river or stream, and to the nearest waterbody recorded on Open Street Map, as contact with unprotected water is a known risk factor for BU [32,53]. Finally, we included a range of human-driven factors which have been associated with BU emergence and transmission: deforestation [54,55], agriculturalization [2,55] and damming of rivers [13,55,56,57]. We calculated Euclidean (straight line) to the nearest area of deforested land and the nearest agricultural area using landcover data [58], and to the nearest dam recorded on Open Street Map [53]. Full details of all variables and their sources are provided in S2 Text.

### Variable selection

We compiled the gridded candidate predictors at a resolution of 5km x 5km within a rectangular area of West Africa from latitude -13.57195, longitude -4.11032, to lat. 16.67107, long. 14.493. This area contained 94% of all BU occurrence locations, 95% of confirmed BU occurrence locations, and all environmental occurrence locations. We extracted the values of predictor variables at the locations of BU cases (all occurrences) and environmental occurrences of *M*. *ulcerans* DNA. We calculated the covariance between all candidate predictors and dropped those which were correlated with another variable with a Pearson correlation coefficient of above 0.8 (or below -0.8), retaining the variable with the strongest existing evidence or biological plausibility for an association with BU or *M*. *ulcerans* distribution or suitability.

### Pseudoabsence and background data

One major challenge in species distribution modelling is the scarcity of data on locations absent for the species or disease of interest, since absence from a given area is difficult to establish with certainty [59]. To account for this, we generated pseudo-absence points, representing the comparator class for the models, in areas where BU was assumed to be absent [60]. We used the surface range envelope function within the *biomod2* package in R [61] to identify areas presumably suitable for the disease (containing values between the 2.5^th^ and 97.5^th^ percentiles of the selected predictor variables) and sampled pseudoabsence points from outside of this envelope. The selection of pseudoabsences was biased to areas with lower evidence of BU endemicity, using data from a systematic review of the geographical distribution of BU [9] to ensure higher coverage of pseudoabsence points in countries with lower evidence for BU. Further details are given in S1 Text.

Another challenge in species distribution modelling is that data from surveys and passive surveillance are often geographically biased due to variation in data collection intensity, which can lead to erroneous predictions if this bias is not accounted for [62]. We generated a separate class of model negative points which we refer to as background points. We distinguish background points from pseudoabsence points on the basis that we make no assumption about the occurrence of or suitability for BU or *M*. *ulcerans* at the background locations [60], and simply use these points to balance out the spatial bias in the occurrence points. This process has previously been termed ‘background thickening’ [63]. Background points were sampled at higher density around recorded occurrence points. More details are provided in S1 Text.

Human background and pseudoabsence points were restricted to a minimum distance of 10km from any BU occurrence location, and environmental background and pseudoabsence points were restricted to 10km from any environmental occurrence location. Within the models, pseudoabsence and background points were downweighted by 50% compared to occurrence points. The distributions of pseudoabsence and background points for the Buruli ulcer suitability models are shown in S3 and S4 Figs and those for the *M*. *ulcerans* suitability models in S5 and S6 Figs.

### Ensemble modelling

The selected environmental covariates were used as predictor variables and the occurrence, pseudoabsence and background locations were included as the outcome. We used the *biomod2* package in R [61,64] to implement seven algorithms: generalized linear models (GLM), generalized additive models (GAM), generalized boosted regression models (GBM), artificial neural networks (ANN), multiple adaptive regression splines (MARS), maximum entropy (MaxEnt) and random forest (RF).

Individual model algorithms were each run 20 times with a random sample of 80% of data points, and evaluated with the remaining 20%. For each algorithm we calculated the mean true skill statistic (TSS), the mean percent correctly classified (PCC) and the mean area under the curve (AUC) of the receiver operation characteristic (ROC) [65]. The TSS is a prevalence-independent measure of predictive accuracy, calculated as sensitivity + specificity– 1 and ranging from -1 to 1 with a score of 1 representing perfect agreement between model predictions and data, and values from 0 to -1 representing performance no better than random. The PCC is a measure of accuracy, calculated as the proportion of points that were correctly classified. The AUC is another measure of model accuracy, measured from 0 to 1 with high values indicating better differentiation of positive and negative values. The AUC is calculated as the area under the curve of the ROC- a plot showing the true positive rate on the y-axis and the false positive rate on the x-axis.

Models with mean AUC above 0.8 were integrated in an ensemble using committee averaging to attribute higher weight to better performing models.

We plotted the importance values representing the contribution of each variable to the model and created marginal effect plots for the modelled covariates in the highest performing model ensemble.

### Estimating total population living in suitable areas

We calculated the total area suitable for BU, *M*. *ulcerans*, and the total area suitable for both, and extracted estimates of the population living in each of these areas from a raster (gridded) dataset representing estimated number of people per 1km^2^ grid square in 2020 [66].

## Results

### Datasets of BU occurrence in humans and *M*. *ulcerans* DNA detection in the environment

**Fig 1:**
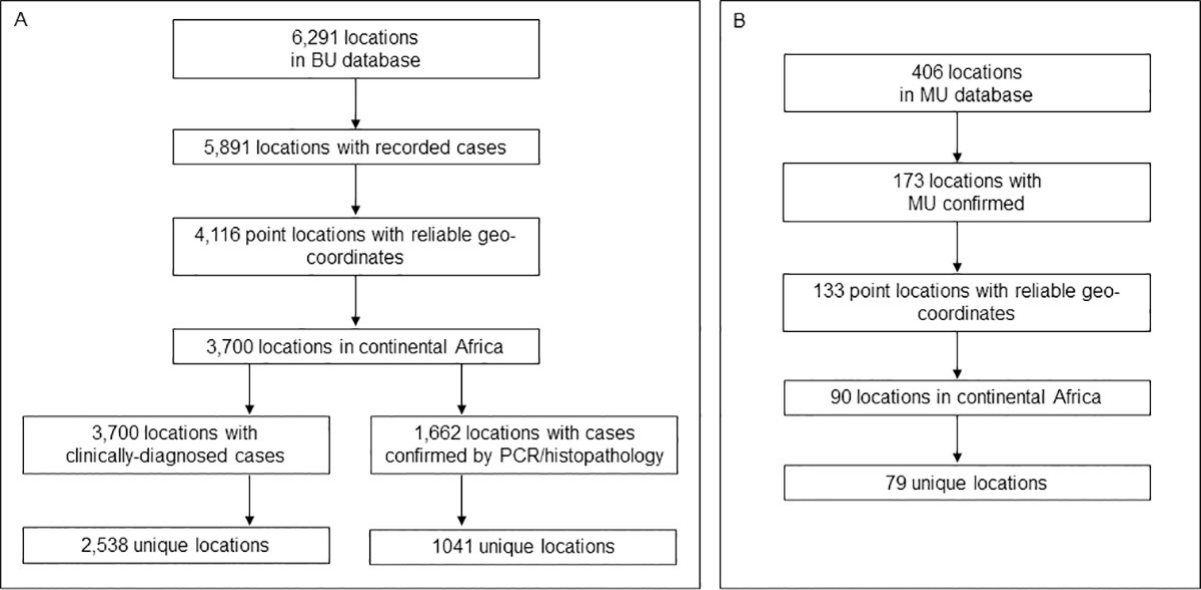
Selection of model occurrence points from Buruli ulcer database. Selection is shown separately for Buruli ulcer occurrences (A) and environmental occurrences of *Mycobacterium ulcerans* DNA.

The modelled data included 3,700 unique point locations with reported cases of BU in Africa (Fig 1A). BU was confirmed by PCR or histopathology at 1,041 unique locations (Fig 1A). There were 79 unique locations where *M*. *ulcerans* DNA had been detected by MIRU, VNTR or qPCR (Fig 1B).

**Fig 2:**
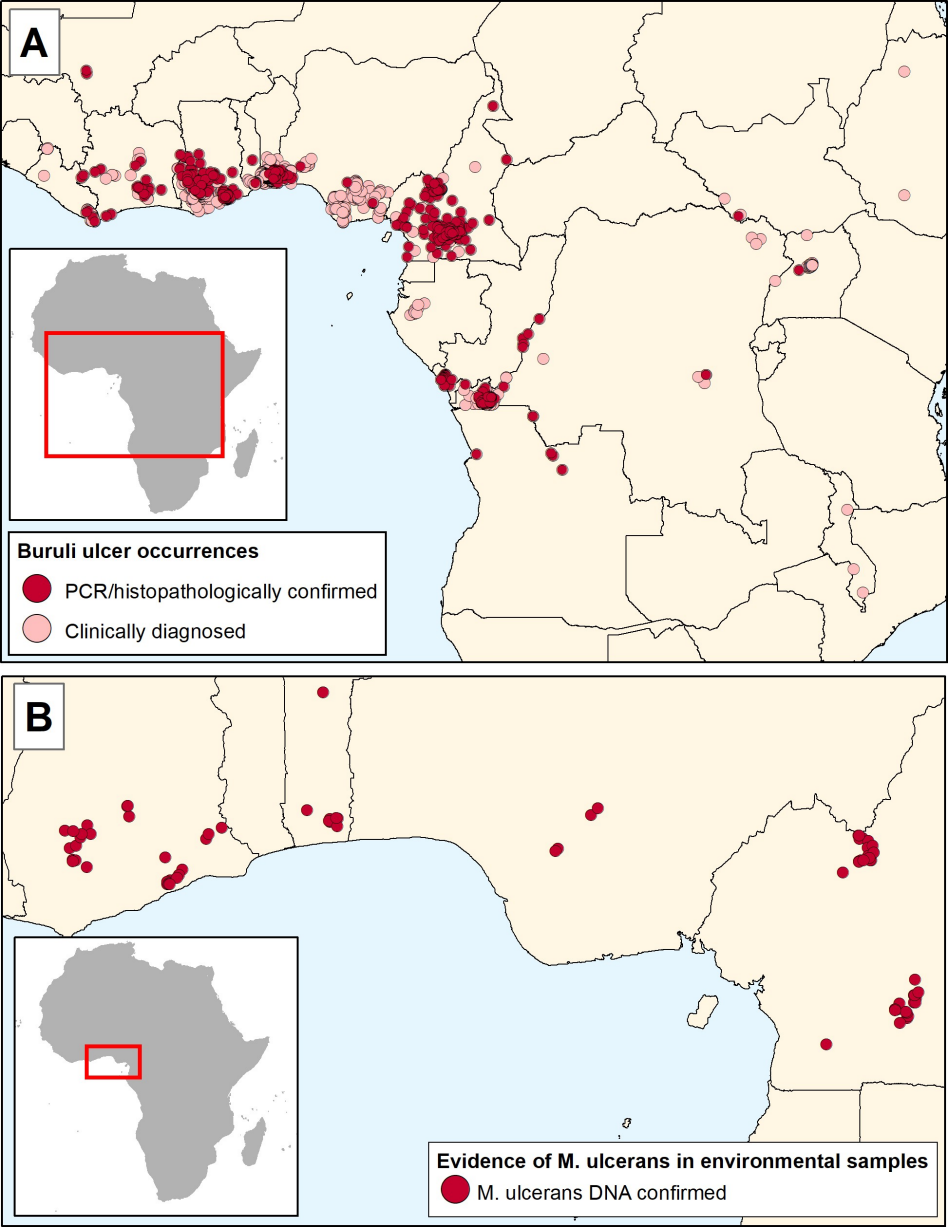
**Distribution of occurrence records for environmental modelling of Burli ulcer (BU) and *Mycobacterium ulcerans* (MU)** (A) Pink dots represent origins of clinically-diagnosed BU cases, red dots represent confirmed cases. (B) Red dots show locations where *M*. *ulcerans* DNA has been isolated from environmental samples and distinguished from DNA from other mycobacteria by multiplex qPCR, or by variable nucleotide tandem repeat, or mycobacterial interspersed repetitive unit typing. All maps were produced in ArcMap 10.7 (ESRI Inc., Redlands CA, USA).

The dataset of clinically diagnosed human cases represented 16 countries, mostly in West and Central Africa, with a few in East and southeast Africa. The confirmed cases were restricted to 12 countries in Africa. The distribution of modelled occurrence points is shown in Fig 2. The time period of human case detection was from 1957 to 2019. The median year of diagnosis was 2010. The 91 records of environmental detection of *M*. *ulcerans* represented four countries: Ghana, Cameroon, Benin and Togo, and covered the period from 2006 to 2018 with a median year of detection of 2013.

### Environmental covariates

Maximum temperature and elevation were excluded from the framework for BU modelling as they were collinear with minimum temperature. The aridity index was dropped as it was colinear with precipitation in the wettest quarter. The topographic wetness index was excluded after the initial modelling step as it made a very limited contribution to the models. The model predictors were therefore annual potential evapotranspiration, minimum temperature, precipitation seasonality, precipitation in the wettest quarter, precipitation in the driest quarter, enhanced vegetation index and distances to rivers and streams, water bodies, dams, deforested areas, and agricultural land.

Maximum temperature, elevation and aridity index were also dropped from the *M*. *ulcerans* modelling framework due to collinearity with minimum temperature. Precipitation seasonality was dropped due to collinearity with precipitation in the driest quarter, and precipitation in the wettest quarter was dropped due to collinearity with minimum temperature. The model predictors were annual potential evapotranspiration, minimum temperature, precipitation in the wettest quarter, enhanced vegetation index, topographic wetness index and distances to rivers and streams, water bodies, dams, deforested areas, and agricultural land.

### Environmental suitability for BU

**Fig 3:**
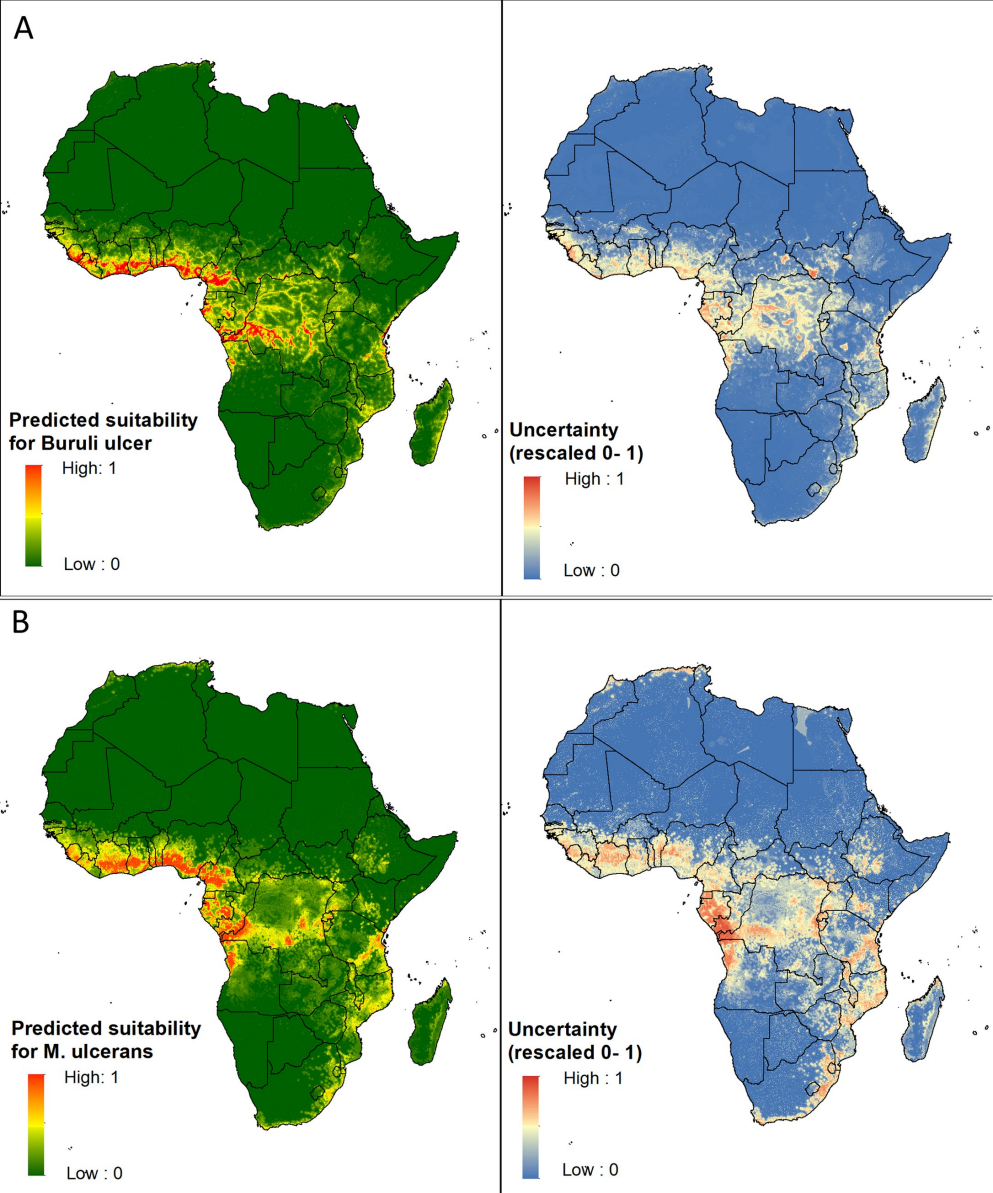
A.) Predicted environmental suitability for the occurrence of BU disease and associated error of prediction. B.) Predicted environmental suitability for the occurrence of *M*. *ulcerans* in the environment and associated error of prediction. All maps were produced in ArcMap 10.7 (ESRI Inc., Redlands CA, USA).

The overall predicted distribution was constrained to humid tropical areas and local scale variation appeared to be driven by hydrological features (Fig 3A). The total area predicted to be suitable for BU was 373,625 km^2^, and the total population living in areas predicted suitable was 72.3 million (Table 2). Pockets of suitability for BU were predicted in 19 countries in Africa, including all 14 countries along the west-central African coastline from Guinea to Angola (S1 Maps and S1 Table). Democratic Republic of the Congo had the widest area predicted suitable, followed by Cameroon. Nigeria had the largest population at risk, with 25.4 million predicted to be living in areas suitable for BU, followed by the Democratic Republic of the Congo where 14.6 million were predicted to be living in suitable areas (S1 Table).

The model including all cases of BU (S7 Fig) gave similar results to the model including confirmed cases only. The Pearson coefficient of correlation between the two models was over 0.95.

All individual distribution models performed well with AUC values above 0.8 (S8 and S9 Figs). Mean PCC scores were between 77.4 and 92.9% and mean TSS scores were between 0.57 and 0.83. RF models performed best with a mean PCC of 92.9%, a mean TSS of 0.83 and mean AUC 0.97. The final ensemble model showed an overall mean AUC of 0.96 with sensitivity of 91.0% and specificity of 88.7%. The mean TSS was 0.79 and the mean kappa score was 0.80 (Table 1).

**Table 1:** Validation metrics for ensemble models for BU and *M*. *ulcerans* suitability.

		Mean	Lower CI	Upper CI
BU suitability	TSS	0.783	0.793	0.796
	AUC	0.964	0.964	0.965
	kappa	0.795	0.788	0.795
MU suitability	TSS	0.867	0.867	0.879
	AUC	0.983	0.983	0.984
	kappa	0.866	0.866	0.873

Distance to the nearest water body was the strongest contributor to the RF models, explaining 23.8% of variance in the model, followed by potential evapotranspiration, which contributed 19.3% of the variance (S10 Fig). Suitability for BU was highest in areas within 10km of the nearest waterbody, and was limited in areas more than 30km from a waterbody (S11 Fig). Suitability was highest in environments with relatively low potential evapotranspiration (1,200–1,600 mm/month), which correlates with the tropical savanna climate zone [67,68].

### Environmental suitability for *M*. *ulcerans*

The GAM, GBM, MARS and RF models performed well with AUC above 0.8 (S12 and S13 Figs), while the GLM, ANN and MAXENT Phillips models performed less well and were excluded from the ensemble. Mean PCC varied from 0.72–0.83 between models and mean TSS was between 0.34 and 0.66. RF outperformed other algorithms in predicting the occurrence of *M*. *ulcerans*. The final ensemble model had a mean TSS score of 0.87, with a sensitivity of 92.4 and specificity of 94.4% (Table 1). The mean AUC was 0.98 and the mean kappa score was 0.87.

Distance to deforested areas and potential evapotranspiration were the strongest predictors of *M*. *ulcerans* occurrence in the RF models, accounting for 28.4% and 28.2% of all variance in the model respectively (S14 Fig). Suitability was predicted to be low in areas more than 30km from any deforested land, and in areas with potential evapotranspiration of >1500mm/month (corresponding to more humid regions characterised by higher rainfall and semi-deciduous to tropical forest) (S15 Fig).

The total area predicted to be suitable for *M*. *ulcerans* was 388,050km^2^, and the total population living in areas predicted suitable was 77.0 million (Table 2). Pockets of suitability were predicted in 17 countries (S1 Table). Nigeria had the widest area predicted suitable (85,350km^2^) followed by Cameroon (66,300km^2^). The highest population living in suitable areas was in Nigeria (33.1 million).

**Table 2:** Total area predicted suitable and population in areas at risk for Buruli ulcer, M. ulcerans, and both, in Africa.

	Total area suitable (km^2^)	*Lower bound*	*Upper bound*	Population in suitable areas	*Lower bound*	*Upper bound*
BU	373,625	*283*,*275*	*498*,*550*	72,341,372	*55*,*617*,*280*	*90*,*689*,*787*
MU	388,050	*265*,*375*	*556*,*225*	77,026,709	*63*,*307*,*468*	*93*,*791*,*018*
BU & MU	163,225	*104*,*575*	*245*,*675*	46,120,259	*34*,*963*,*000*	*58*,*963*,*221*

Suitability for BU and *M*. *ulcerans* is shown by country in S1 Maps.

### Overlap of suitability for BU and *M*. *ulcerans*

**Fig 4:**
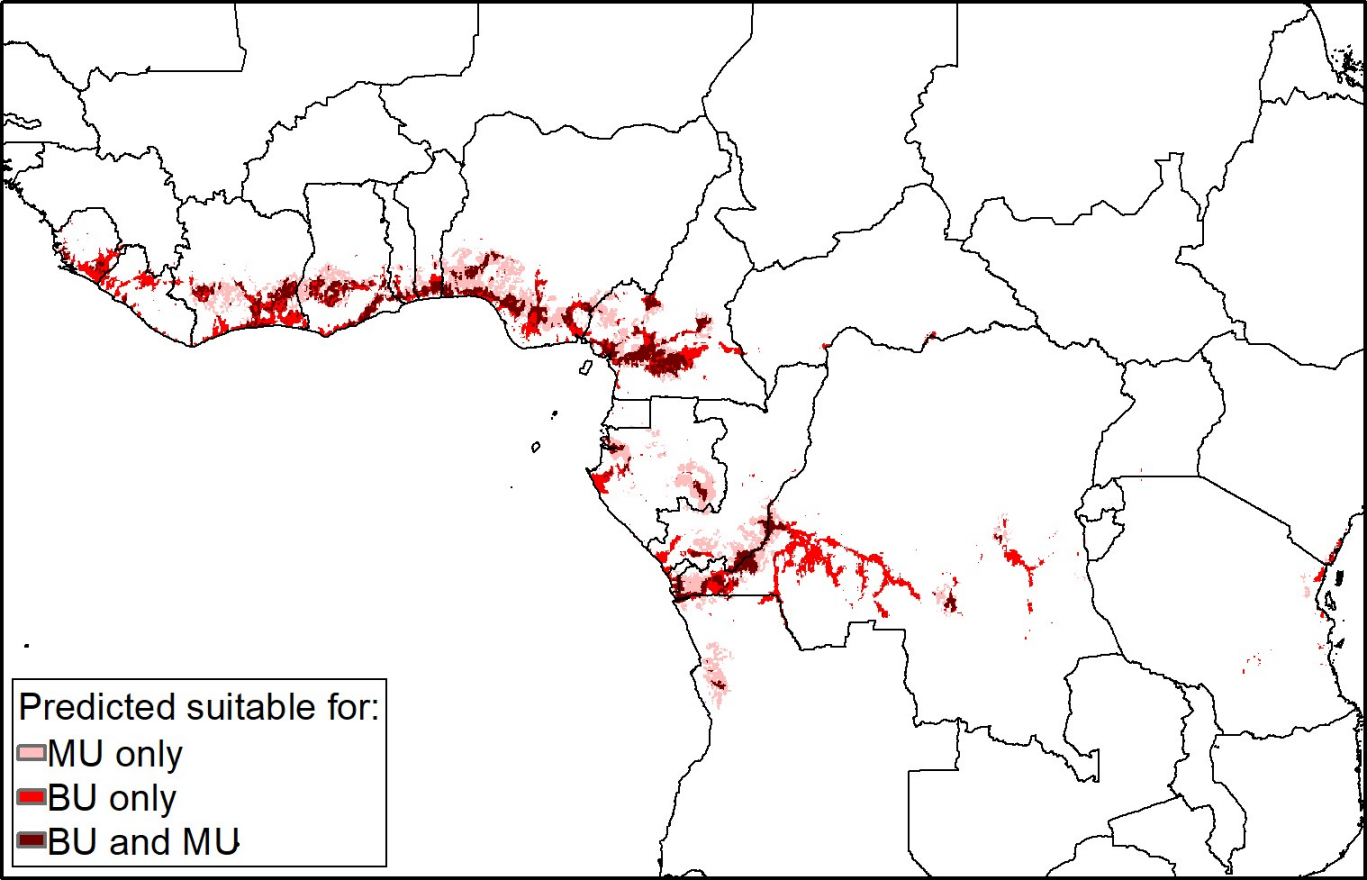
Predicted overlap of environmental suitability for BU and of M. ulcerans occurrence. Pink colour represents areas where Mycobacterium ulcerans (MU) is predicted to occur based on the optimal threshold of environmental suitability (0.56) but where Buruli ulcer (BU) is not predicted. Red represents areas where BU is predicted based on the optimal threshold of environmental suitability (0.51) but MU is not. Both BU and MU are predicted to occur in areas shown in dark red. All maps were produced in ArcMap 10.7 (ESRI Inc., Redlands CA, USA).

The total area predicted to be suitable for both BU and *M*. *ulcerans* was 163,225km^2^, with 46.1 million people predicted to be living in areas at risk. There were some differences in the extents of the areas predicted suitable for BU disease and environmental *M*. *ulcerans* (Fig 4). There were wide areas predicted suitable for *M*. *ulcerans* but not for BU disease, mostly located around the periphery of known endemic foci in west African countries. There were patches of predicted suitability for BU but not *M*. *ulcerans* in DRC, Sierra Leone, Liberia and other countries in West Africa. The highest populations living in areas predicted suitable for both BU and *M*. *ulcerans* were in Nigeria and DRC, with 18.0 and 10.1 million respectively at risk.

## Discussion

We have used ecological niche modelling to identify environmental factors associated with the occurrence of Buruli ulcer and its causative agent *M*. *ulcerans*, and to predict environmental suitability for the disease and bacterium across continental Africa. Incorporating existing data on BU distribution at multiple spatial levels and a set of relevant environmental covariates, the resulting maps represent evidence-based predictions which are intended to guide future surveillance for BU.

The model predictions were broadly consistent with the recognised distribution of BU in Africa [9]. We identified pockets of suitability for BU in patchy foci throughout the known-endemic range of the disease, particularly in the tropical zones of countries around the Gulf of Guinea. Suitability was also predicted in a number of regions not previously recognised as endemic, particularly in Sierra Leone, the north-west coast of Liberia, and parts of southern Nigeria. Wide areas of suitability were predicted beyond the known foci of BU in DRC, particularly along the Kasai river basin in the central-west region of the country. In Gabon, an extended focus of suitability was predicted towards the mouth of the Ogooue River. Several cases of BU have been reported from this area [69], but were not included in the main model presented here as they were not confirmed by PCR or histopathological analysis. A further Gabonese focus was predicted in-land, in the south east of the country, which has no previous evidence of cases. Restricted foci of suitability were predicted in Equatorial Guinea, corresponding to the origin of cases diagnosed by an expert in BU between 1995 and 2005 [70,71], although the country has no evidence of cases reported in peer-reviewed literature.

There were also regions predicted unsuitable by the models where empirical evidence suggests previous cases. There are several possible reasons for these discrepancies. Some locations in northern Cameroon with evidence of PCR-confirmed BU were found to be unsuitable for the disease. Given the great volume of surveillance data collected by the well-established BU control programme in Cameroon, some patients are likely to have been diagnosed outside the region where they acquired the disease [72], and we consider it plausible that some regions where BU has been recorded are not actually suitable for transmission. The model did not predict occurrence of BU or *M*. *ulcerans* within the early BU foci in Uganda and northern DRC [46,73–75], or in South Sudan where cases were reported in the early 2000’s [76], although moderate suitability was predicted around these areas. This discrepancy may indicate that these foci were associated with transient factors which are no longer locally prevalent, or that the model lacks sensitivity in areas with sparse occurrence points. The fact that these models do not include a temporal component limits their usefulness for understanding drivers of the emergence (and disappearance) of BU. Since the number of geo-located confirmed occurrences and availability of data on spatial covariates prior to 1991 was limited, we were not able to stratify the analysis by time period.

Cases of BU have recently been reported in Senegal [77,78] and Madagascar [79], where occurrence was not predicted by the model. Assuming these recent cases represent true instances of autochthonous transmission of *M*. *ulcerans*, this demonstrates a limitation of these models in their ability to predict emergent foci in regions that are environmentally distinct from known-endemic areas. Incorporating new data, particularly those originating from new-endemic or newly recognised endemic areas, will help to improve the generalisability of the models in the future.

Although we intend these models to be used as predictive rather than explanatory tools, the environmental associations we identified have relevance to understanding the ecological niche and transmission of *M*. *ulcerans*. We emphasise that the covariates we included should be viewed as associated, rather than causal factors. Both BU and *M*. *ulcerans* were constrained to tropical climate zones [68] due to sensitivity to potential evapotranspiration, temperature, and precipitation indicators. These findings fit with the current understanding of the distribution of BU in Africa and support evidence for a different epidemiology of the disease in Africa compared to endemic areas of temperate Australia and Japan [80]. Previous evidence suggests that the strain of *M*. *ulcerans* which causes BU in Japan may be adapted to cooler climates [80], while in Australia there is evidence for an important role of terrestrial mammals [81]. The existence of mammalian reservoirs may enable the disease to emerge in climates which are unfavourable for maintenance of bacterial populations in the abiotic environment. Importantly, this does not rule out the possibility of an animal reservoir for BU in [82] Africa [83], since the range of suitability predicted by these models may illustrate the ecological niche of a different reservoir taxon.

We identified a number of human-influenced variables as predictors of *M*. *ulcerans* occurrence, and to a lesser extent, BU occurrence. Variables such as distance to deforested areas, dams, and agricultural land, and the enhanced vegetation index are expected to show greater temporal variation than bioclimatic factors, and as such may be more relevant to understanding drivers of change in the distribution of BU. Environmental disturbance has been postulated as a driver of BU emergence [84], and higher rates of disease have been reported in agricultural areas on the peripheries of forests [85]. Local-scale variation in these factors resulted in a patchy distribution of predicted suitability, consistent with our understanding of the epidemiology of BU, which is recognised to be highly focal in endemic settings [86].

Although the models we developed were designed to represent the ecological niche of *M*. *ulcerans* and BU, many aspects of the ecology and transmission of the bacterium were not represented. The models we developed were ‘black-box’ type representations which risk oversimplifying the process of disease transmission as they do not account for ecological complexities including the behaviour and demography of hosts and interactions between host species [87]. Since these components of BU transmission are currently not well understood, we were limited to assuming that the observed occurrences of *M*. *ulcerans* and BU would adequately represent the outcomes of these interactions [87]. However, the more general prediction of suitability has practical applications in informing surveillance efforts, even if it does not enable precise estimation of transmission risk.

The available dataset of locations where *M*. *ulcerans* DNA was detected in the environment was restricted, including only 79 unique locations in four countries, and cannot be expected to represent all environmental conditions where the bacterium occurs. The limited coverage of *M*. *ulcerans* data points is a potential source of bias, since the *M*. *ulcerans* models may be less restrictive than those for BU, potentially explaining the wider predicted occurrence of *M*. *ulcerans*. The scale of analysis (grid cells at 5km x 5km) may have also limited our ability to quantify the effect of predictors varying over small geographical scales and to capture fine scale variation in environmental suitability for BU. The models predicted large contiguous areas of suitability in areas with suitable conditions, particularly in West Africa. Such areas may be suitable in reality, but exhibit an uneven distribution of disease due to factors not included in our models.

Despite these limitations, the suitability maps provide a delineation of areas potentially at risk for BU beyond what is known from the distribution of reported cases, currently the basis for targeting of surveillance and control. Given the recognised scale of underreporting of BU [9], the current approach is likely to exclude cases outside of known disease foci, and we suggest that areas predicted suitable for BU and *M*. *ulcerans* should be considered as targets for case finding activities, with the aim of identifying unrecognised foci and patients not known to the health system. Based on the wide areas of suitability predicted by this work and existing evidence of under-reporting of BU [88], the south of Nigeria would be a key target for case finding activities. The foci predicted in Gabon, Equatorial Guinea and Sierra Leone, associated with limited evidence of previous cases, would also be targets for further investigation. We note however, that predictions in these regions (not represented by occurrences included in the model) were associated with high levels uncertainty, which should be considered in the design of any future surveys.

Using the model predictions to inform the design of cross-sectional surveys for BU could improve the efficiency of such surveys. In a nationwide survey for podoconiosis in Cameroon, the selection of survey communities was stratified according predicted suitability for the disease based on a model trained mainly using data from Ethiopia [89]. This survey identified higher rates of podoconiosis in communities that were predicted suitable, implying a benefit in terms of the cost per case identified, compared to a survey employing random selection of survey communities. Another mechanism to improve cost effectiveness may be to combine the predictions from these models with models for other diseases in order to target integrated surveys for rare outcomes [90].

In conclusion, we have identified areas of high suitability for BU and *M*. *ulcerans* within known endemic-areas, and in areas not currently recognised as endemic, but with evidence of possible undiagnosed or misdiagnosed BU. The population at highest risk of BU is within areas where BU and *M*. *ulcerans* niches overlap, comprising over 46 million people in 2020. The focal nature of BU distribution, the recognised scale of under-detection, and the impact of late diagnosis on disease severity strongly suggest a targeted approach to active case finding as a means to control this disease. The fine-scale, evidence-based predictions presented here could provide a tool to target such efforts, which will improve our understanding of the burden and distribution of the disease and help to increase the proportion of cases linked to treatment.

## Supporting information

S1 Maps Predicted environmental suitability for the occurrence of BU disease and *M*. *ulcerans* in the environment, in countries predicted to be suitable. (PDF)

S1 Text Selection of background and pseudoabsence points. (DOCX)

S2 Text Environmental variables used in modelling, including potential environmental predictors and their sources and the covariates that were included in the models of BU and *M*. *ulcerans* suitability. (DOCX)

S1 Fig Distribution of PCR and histopathologically confirmed BU cases, by year of diagnosis. (TIF)

S2 Fig Distribution of clinically diagnosed BU cases, by year of diagnosis. (TIF)

S3 Fig Selection of pseudoabsence points included in Buruli ulcer suitability models. Pseudoabsence points were selected outside of the BU surface range envelope (white; the area containing values between the 2.5^th^ and 97.5^th^ percentile of all predictor variables) and selection was biased according to the strength of evidence for BU at national or subnational level (yellow to blue shading) using results from Simpson et al. Lancet Glob. Health 2019.
(TIF)

S4 Fig Selection of pseudoabsence points included in *Mycobacterium ulcerans* suitability models. Pseudoabsence points were selected outside of the MU surface range envelope (white; the area containing values between the 2.5^th^ and 97.5^th^ percentile of all predictor variables) and selection was biased according to the strength of evidence for BU and MU at national or subnational level (yellow to blue shading) using results from Simpson et al. Lancet Glob. Health 2019.
(TIF)

S5 Fig Distribution of background points used in Buruli ulcer suitability models. Background points were restricted to a minimum distance of 10km from human occurrence points (not shown on the map) and were selected with probability defined by the kernel density surface representing the density of occurrence points.
(TIF)

S6 Fig Distribution of background points used in *Mycobacterium ulcerans* suitability models. Background points were restricted to a minimum distance of 10km from human or environmental occurrence points (not shown on the map) and were selected with probability defined by the kernel density surface representing the density of occurrence points.
(TIF)

S7 Fig Predicted environmental suitability for the occurrence of BU disease and associated error of prediction, including all clinically diagnosed cases of BU. (TIF)

S8 Fig Individual model performance evaluation statistics for models of environmental suitability for Buruli ulcer. Performance evaluated in terms of the mean true skill statistic (TSS) and the mean area under the curve (AUC) of the receiver operation characteristic.
(TIF)

S9 Fig Individual model performance evaluation statistics for models of environmental suitability for Buruli ulcer. Performance evaluated in terms of accuracy (percent correctly classified) and the mean area under the curve (AUC) of the receiver operation characteristic. Individual model algorithms: ANN = artificial neural networks; GAM = generalized additive models; GBM = generalized boosted regression models; GLM = generalized linear models; MARS = multiple adaptive regression splines; MAXENT. Phillips = maximum entropy; RF = random forest.
(TIF)

S10 Fig Variable importance plots of the contribution of environmental covariates to random forest models of suitability. Shows contribution of variables to model for Buruli ulcer. Blue bars = variables selected as predictors of BU occurrence and *M*. *ulcerans* in the environment. Orange bars = variables selected as predictors of Buruli ulcer (BU) occurrence only.
(TIF)

S11 Fig Marginal effect plots showing the relationship between environmental covariates and suitability for Buruli ulcer and *Mycobacterium ulcerans* in random forest models. Marginal Effect of Environmental Predictors on Environmental Suitability for Buruli ulcer
(TIF)

S12 Fig Individual model performance evaluation statistics for models of environmental suitability for *Mycobacterium ulcerans*. Performance evaluated in terms of the mean true skill statistic (TSS) and the mean area under the curve (AUC) of the receiver operation characteristic.
(TIF)

S13 Fig Individual model performance evaluation statistics for models of environmental suitability for *Mycobacterium ulcerans*. Performance evaluated in terms of accuracy (percent correctly classified) and the mean area under the curve (AUC) of the receiver operation characteristic. Individual model algorithms: ANN = artificial neural networks; GAM = generalized additive models; GBM = generalized boosted regression models; GLM = generalized linear models; MARS = multiple adaptive regression splines; MAXENT. Phillips = maximum entropy; RF = random forest.
(TIF)

S14 Fig Variable importance plots of the contribution of environmental covariates to random forest models of suitability. Shows contribution of variables to model for *Mycobacterium ulcerans*. Blue bars = variables selected as predictors of BU occurrence and *M*. *ulcerans* in the environment Green bars = variables selected as predictors of *M*. *ulcerans* in the environment only
(TIF)

S15 Fig Marginal effect plots showing the relationship between environmental covariates and suitability for Buruli ulcer and *Mycobacterium ulcerans* in random forest models. Marginal Effect of Environmental Predictors on Environmental Suitability for *Mycobacterium ulcerans*. Variables are plotted in order of their contribution to the random forest model. Marginal effect plots illustrate the effect of each explanatory variable on the outcome of suitability for Buruli ulcer. Variables are plotted in order of their contribution to the random forest model. *Interpretation of Enhanced Vegetation Index: low values (0.1–0.15) represent areas of barren rock or sand and built-up land; moderate values (0.15–0.3.5) may indicate shrubs, grassland or cropland; higher values (0.35–0.6) may indicate mixed wood and shrubs or open forest.
(TIF)

S1 Table Total area predicted suitable and population living in suitable areas for Buruli ulcer, *M*. *ulcerans*, and both, by country in African continent. WM = weighted mean prediction across final ensemble model; LB = lower bound of prediction; UB = upper bound of prediction BU = Buruli Ulcer; MU = Mycobacterium ulcerans; CAR = Central African Republic; DRC = Democratic Republic of the Congo
(XLSX)
