# Supplementary material for: Mapping suitability for Buruli ulcer at fine spatial scales across Africa: A modelling study
Source: PLoS Negl Trop Dis. 2021 Mar 3;15(3):e0009157. doi: 10.1371/journal.pntd.0009157 (PMC7959670; doi:10.1371/journal.pntd.0009157)
Supplement: S1 Maps — (PDF) [file pntd.0009157.s001.pdf]

Angola

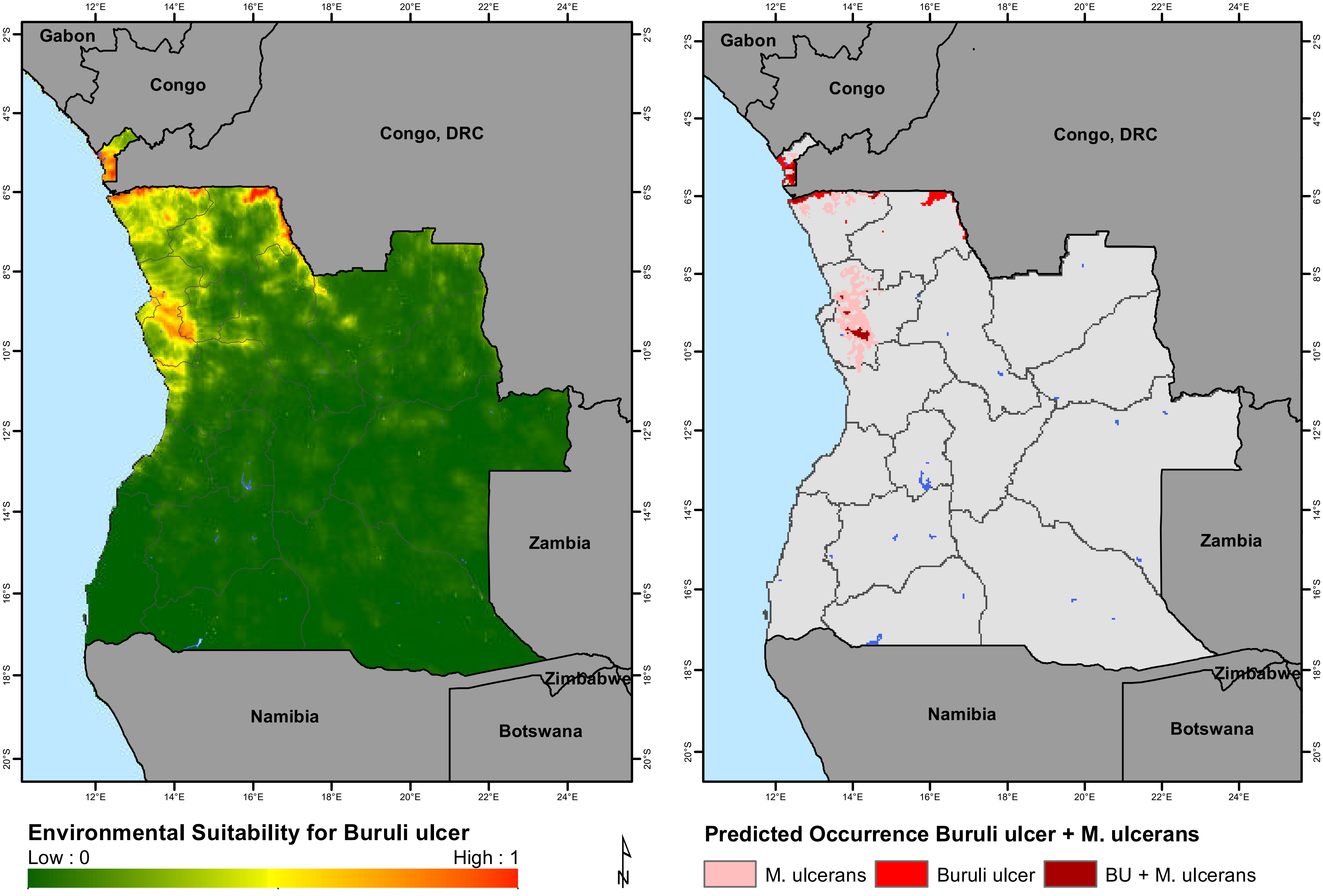

Gabon
Congo
Congo, DRC
Zambia
Zimbabwe
Namibia
Botswana
12°E
14°E
16°E
18°E
20°E
22°E
24°E
2°S
4°S
6°S
8°S
10°S
12°S
14°S
16°S
18°S
20°S
Environmental Suitability for Buruli ulcer
Low : 0
High : 1
N
Predicted Occurrence Buruli ulcer + M. ulcerans
M. ulcerans
Buruli ulcer
BU + M. ulcerans

Benin

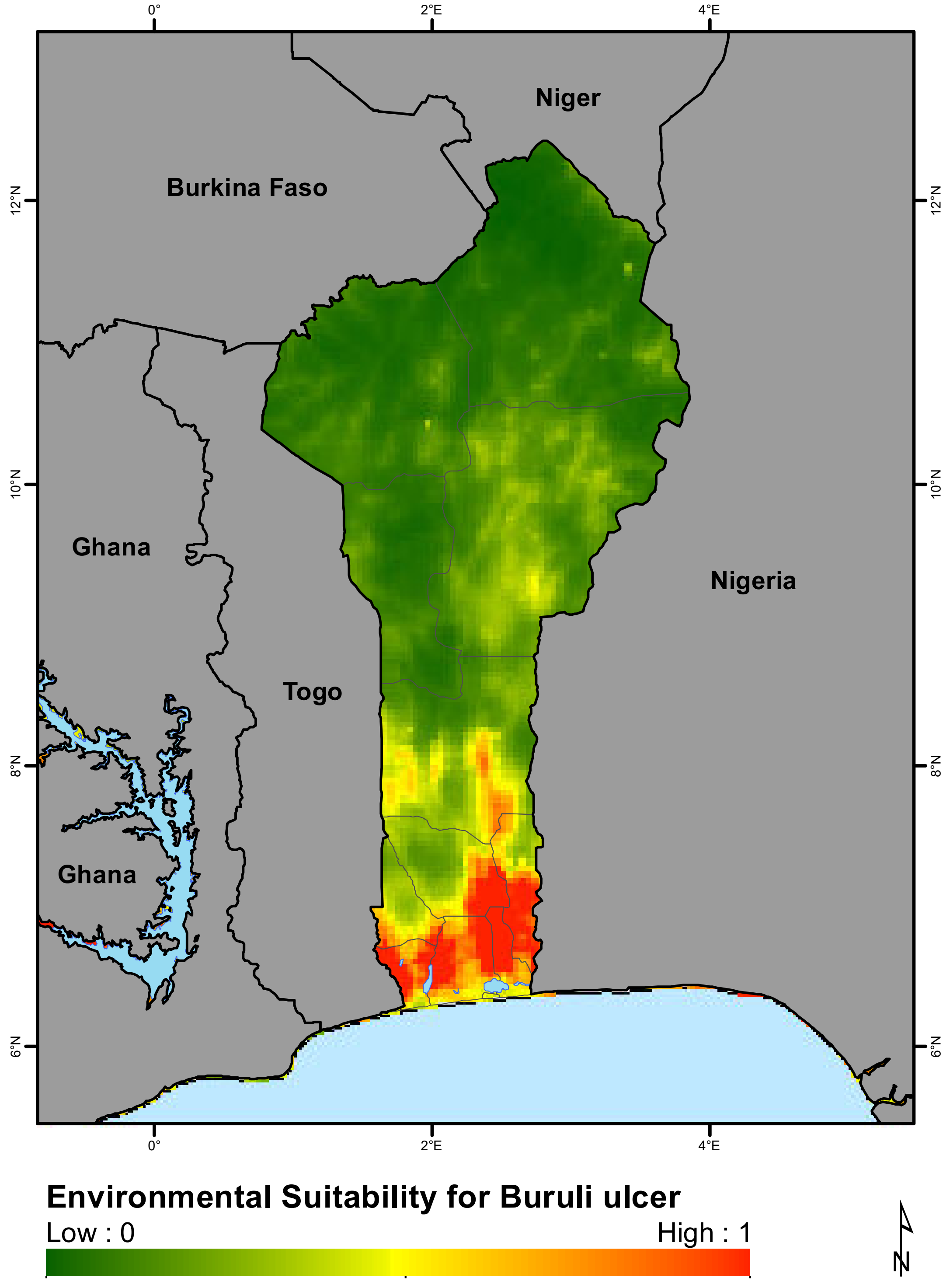

0°
2°E
4°E
12°N
10°N
8°N
6°N
Niger
Burkina Faso
Ghana
Nigeria
Togo
Ghana
Environmental Suitability for Buruli ulcer
Low : 0
High : 1

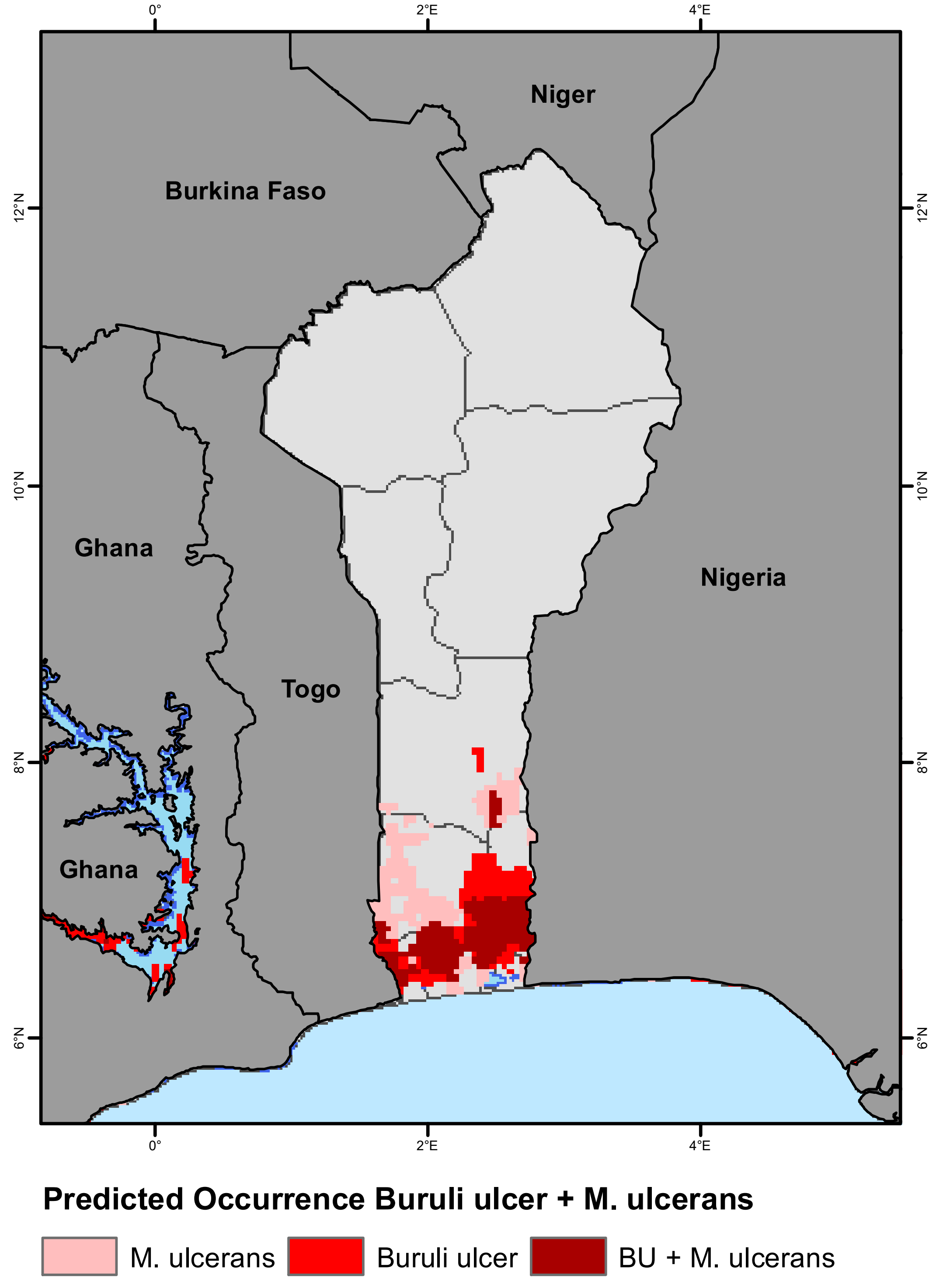

0°
2°E
4°E
12°N
10°N
8°N
6°N
Niger
Burkina Faso
Ghana
Nigeria
Togo
Ghana
N
Predicted Occurrence Buruli ulcer + M. ulcerans
M. ulcerans
Buruli ulcer
BU + M. ulcerans

Burundi

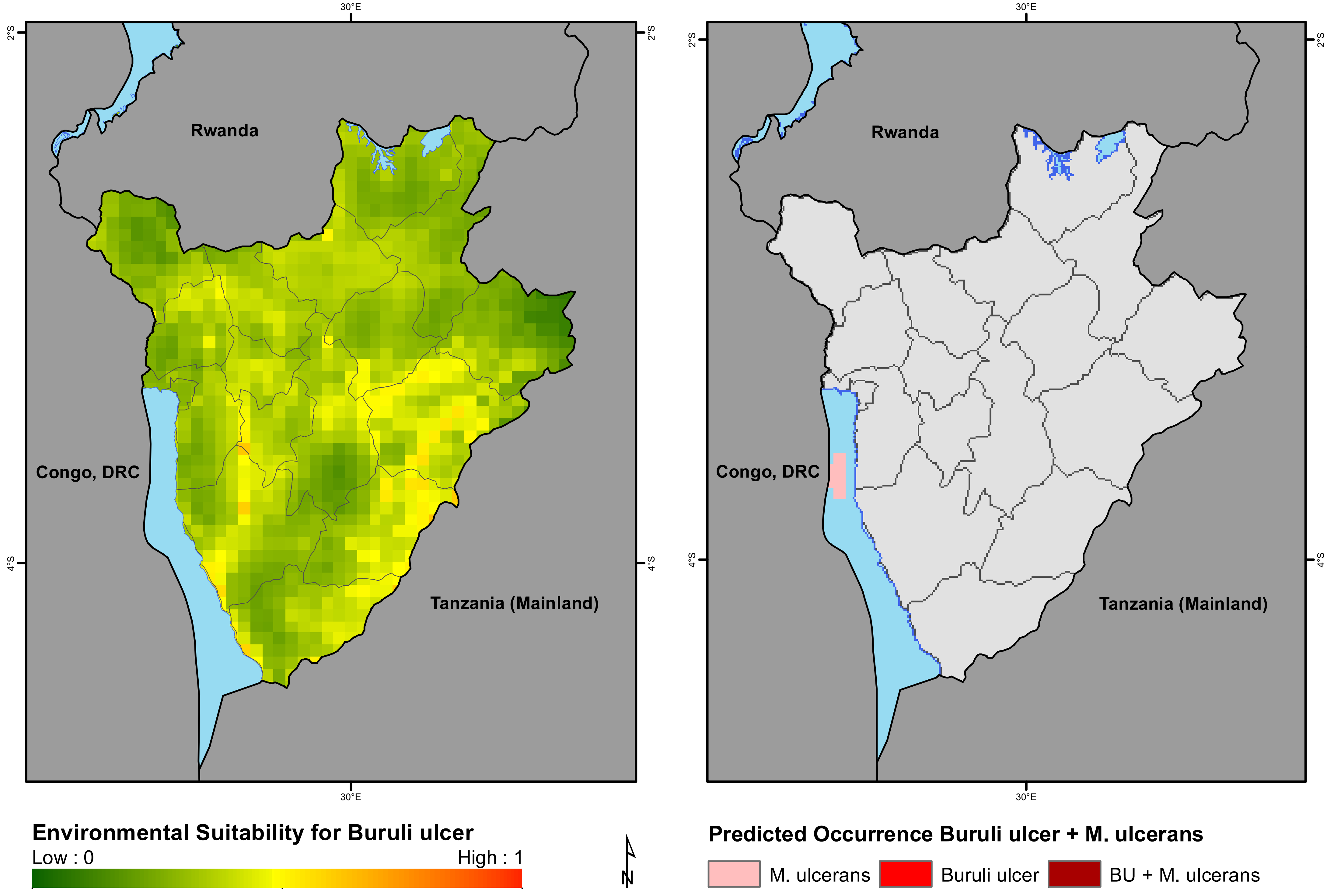
30°E
2°S
4°S
Rwanda
Congo, DRC
Tanzania (Mainland)
Environmental Suitability for Buruli ulcer
Low : 0
High : 1
N
Predicted Occurrence Buruli ulcer + M. ulcerans
M. ulcerans
Buruli ulcer
BU + M. ulcerans

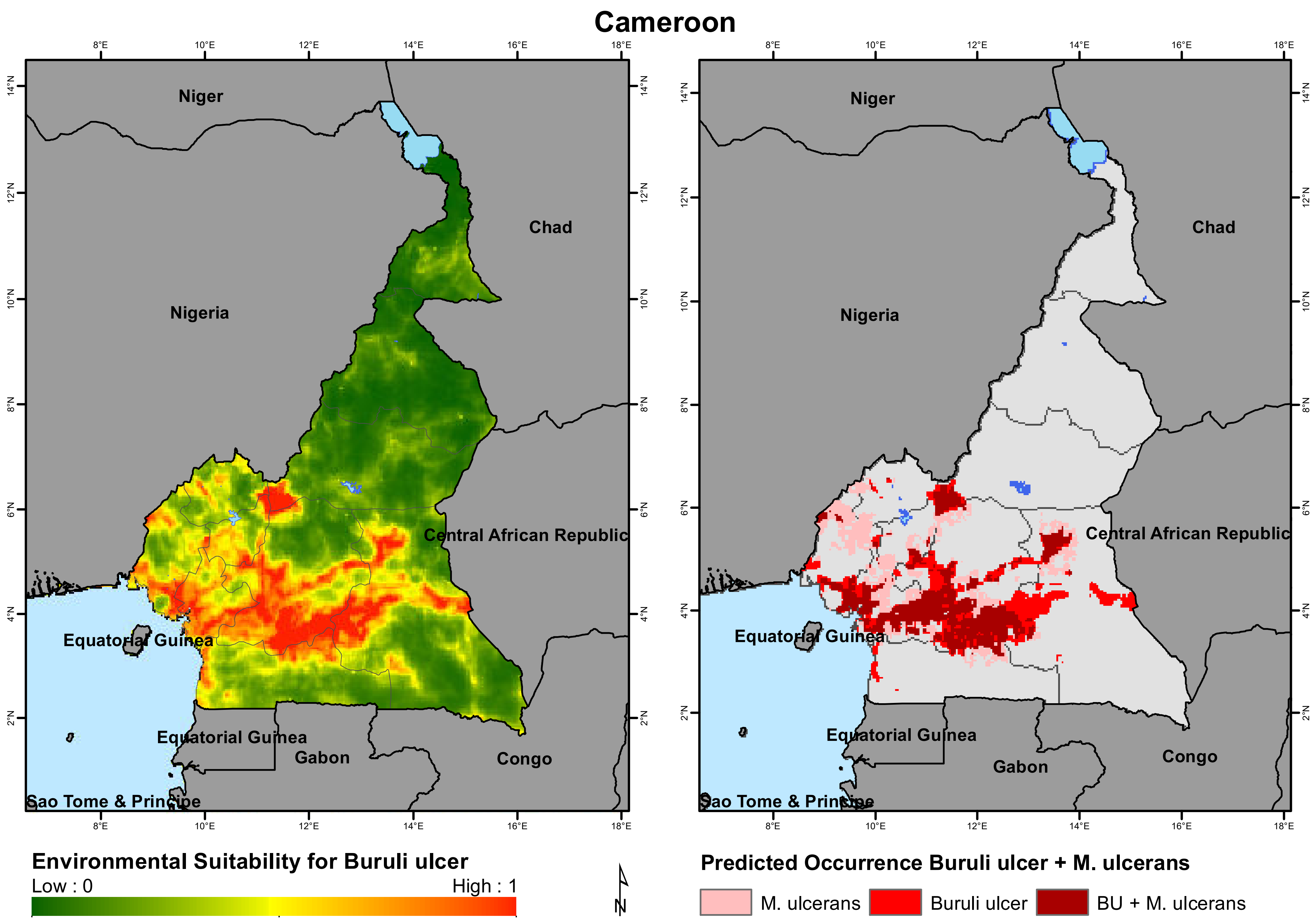
Cameroon
Niger
Nigeria
Chad
Central African Republic
Equatorial Guinea
Equatorial Guinea
Gabon
Congo
Sao Tome & Principe
8°E
10°E
12°E
14°E
16°E
18°E
2°N
4°N
6°N
8°N
10°N
12°N
14°N
Environmental Suitability for Buruli ulcer
Low : 0
High : 1
N
Predicted Occurrence Buruli ulcer + M. ulcerans
M. ulcerans
Buruli ulcer
BU + M. ulcerans

Central African Republic

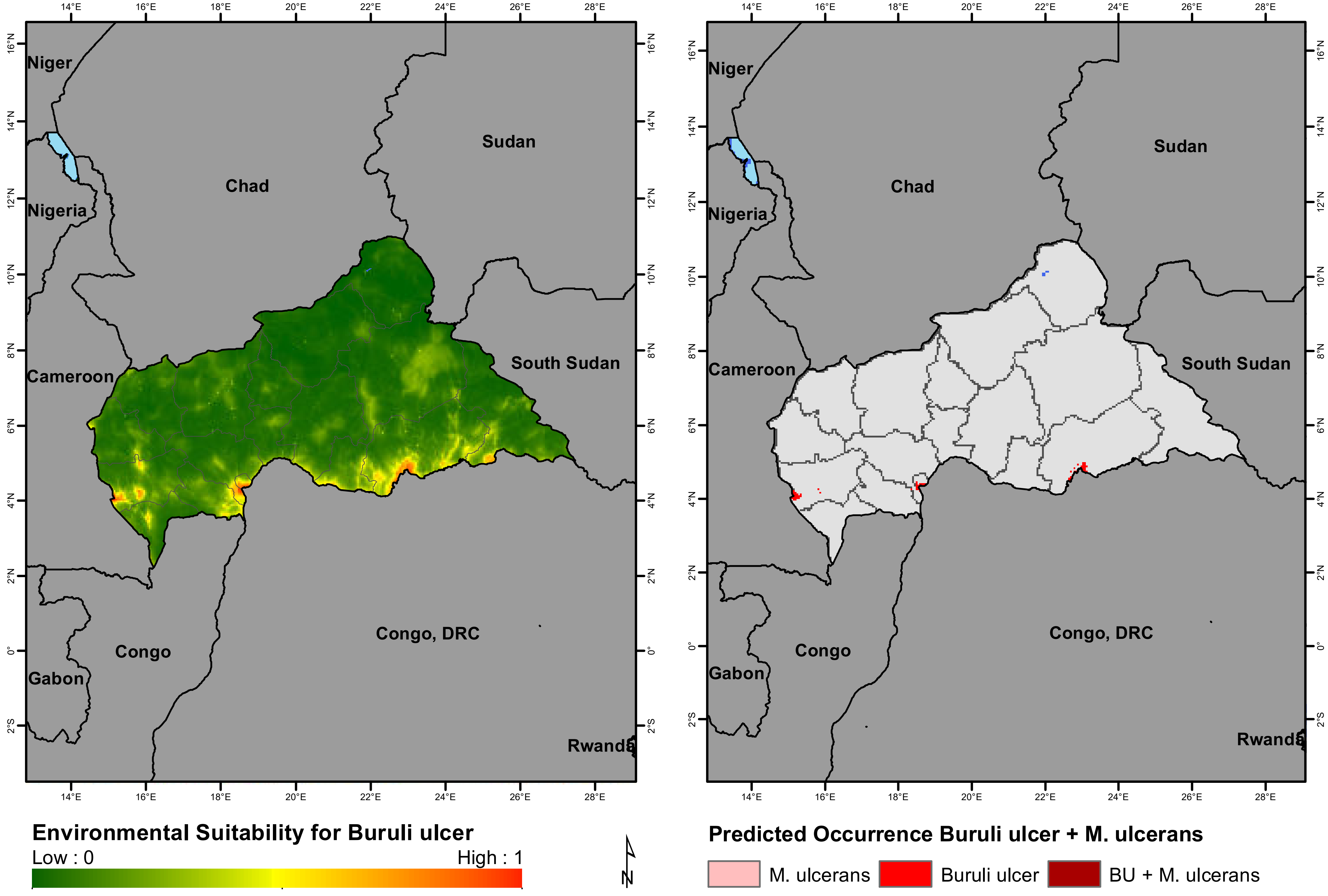

Congo

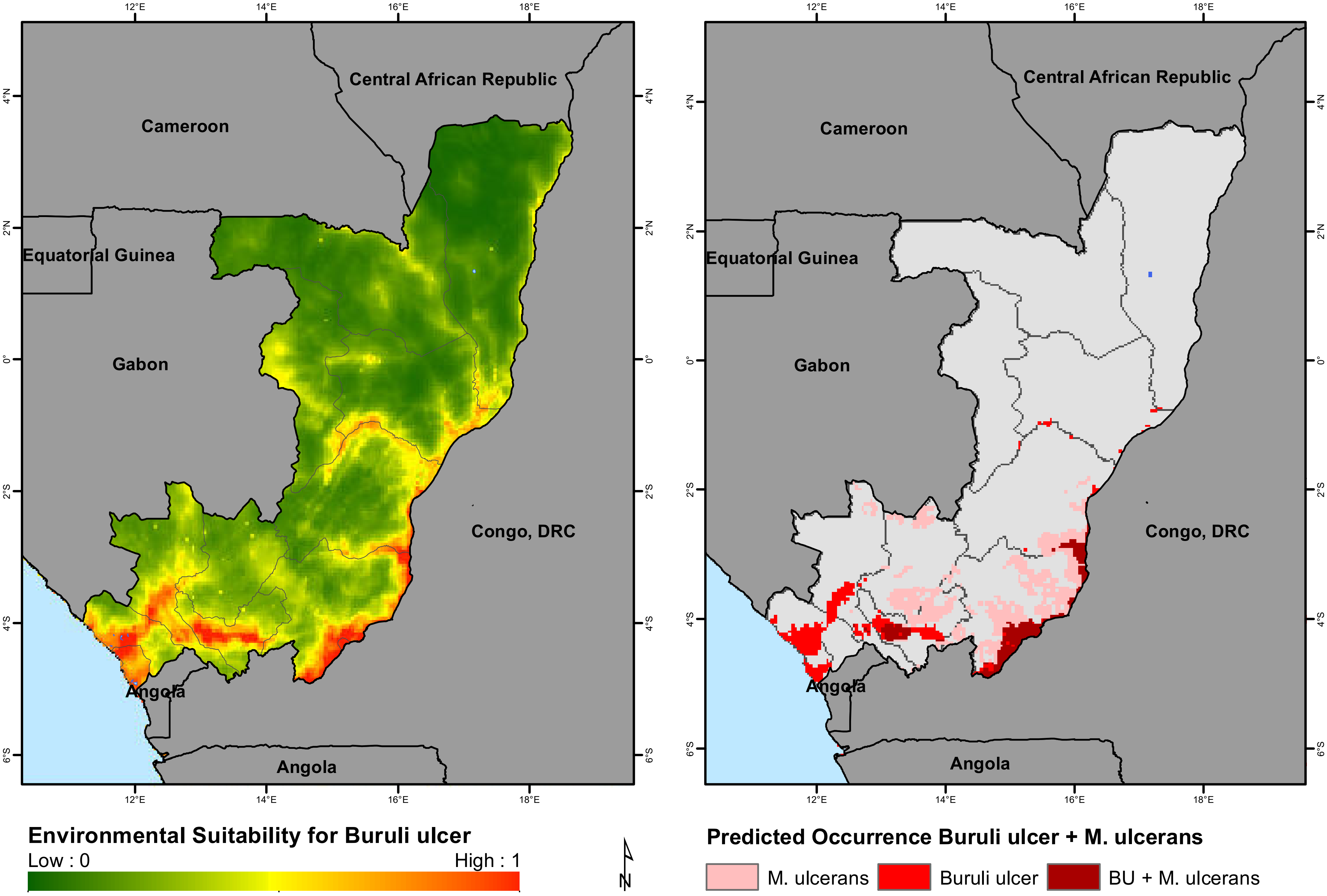
12°E
14°E
16°E
18°E
4°N
2°N
0°
2°S
4°S
6°S
Central African Republic
Cameroon
Equatorial Guinea
Gabon
Congo, DRC
Angola
Angola
Environmental Suitability for Buruli ulcer
Low : 0
High : 1
N
Predicted Occurrence Buruli ulcer + M. ulcerans
M. ulcerans
Buruli ulcer
BU + M. ulcerans

Congo, DRC

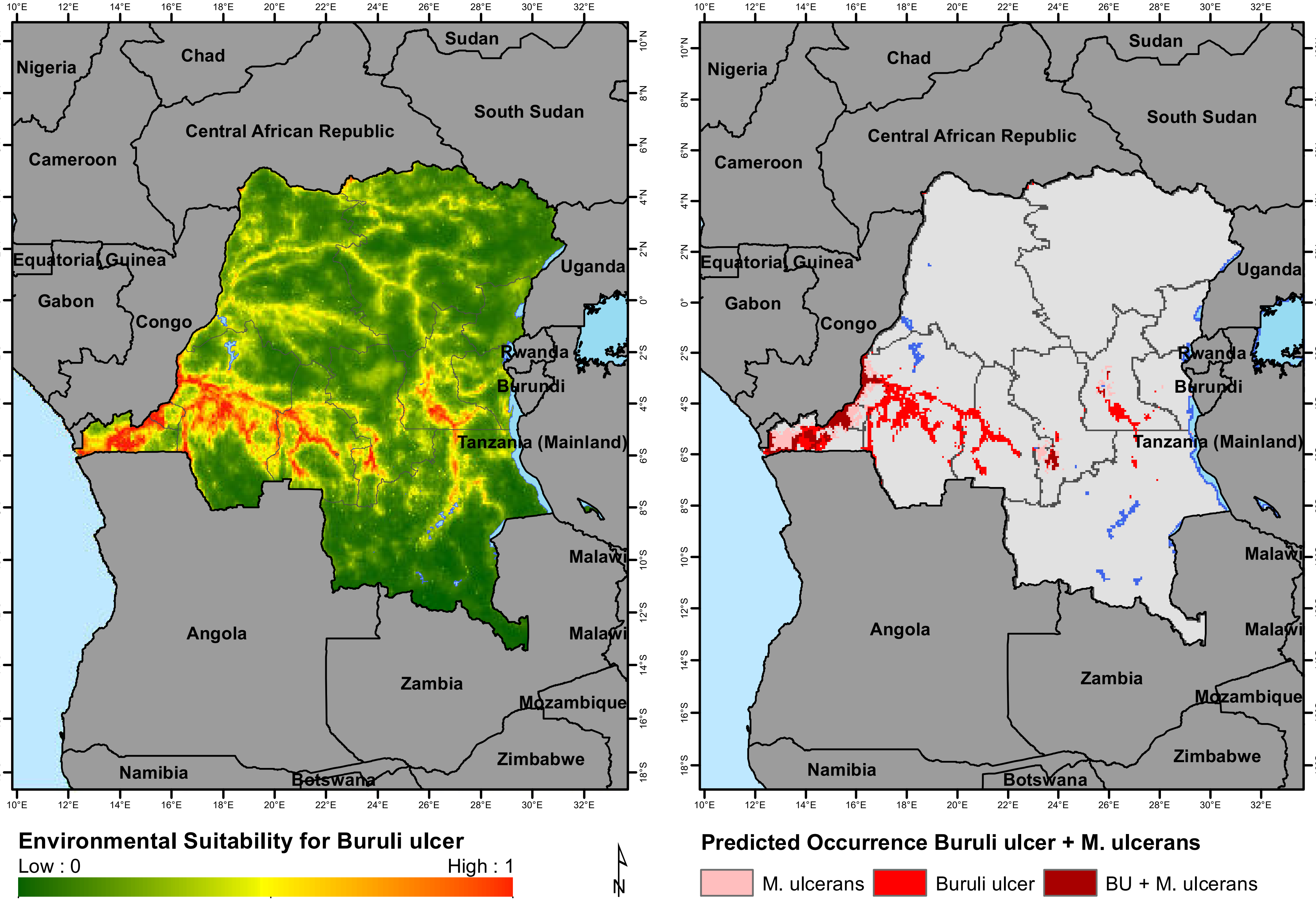
Nigeria
Chad
Sudan
South Sudan
Central African Republic
Cameroon
Equatorial Guinea
Gabon
Congo
Uganda
Rwanda
Burundi
Tanzania (Mainland)
Malawi
Angola
Zambia
Mozambique
Zimbabwe
Namibia
Botswana
Environmental Suitability for Buruli ulcer
Low : 0
High : 1
N
Predicted Occurrence Buruli ulcer + M. ulcerans
M. ulcerans
Buruli ulcer
BU + M. ulcerans

Cote d'Ivoire

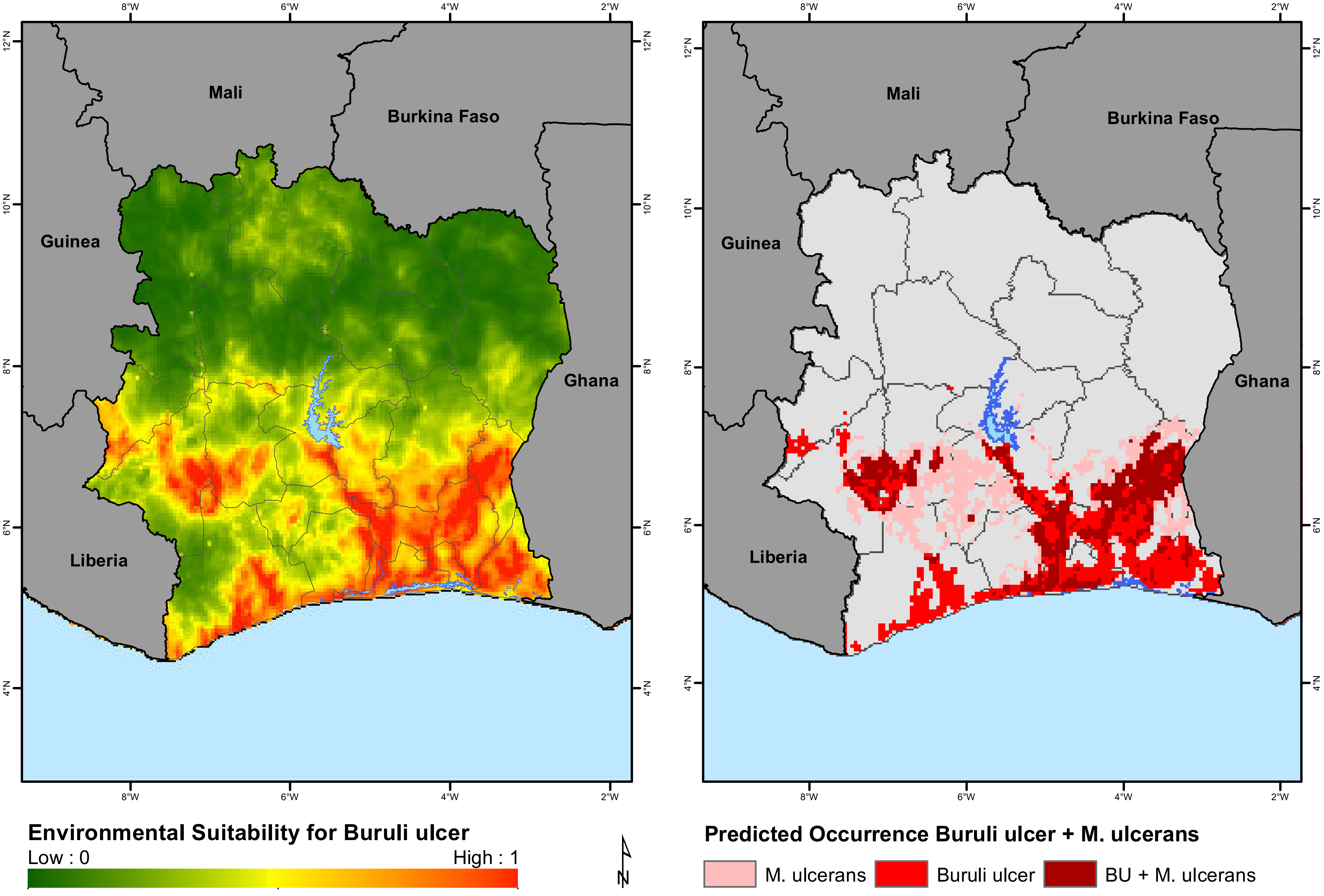

Equatorial Guinea

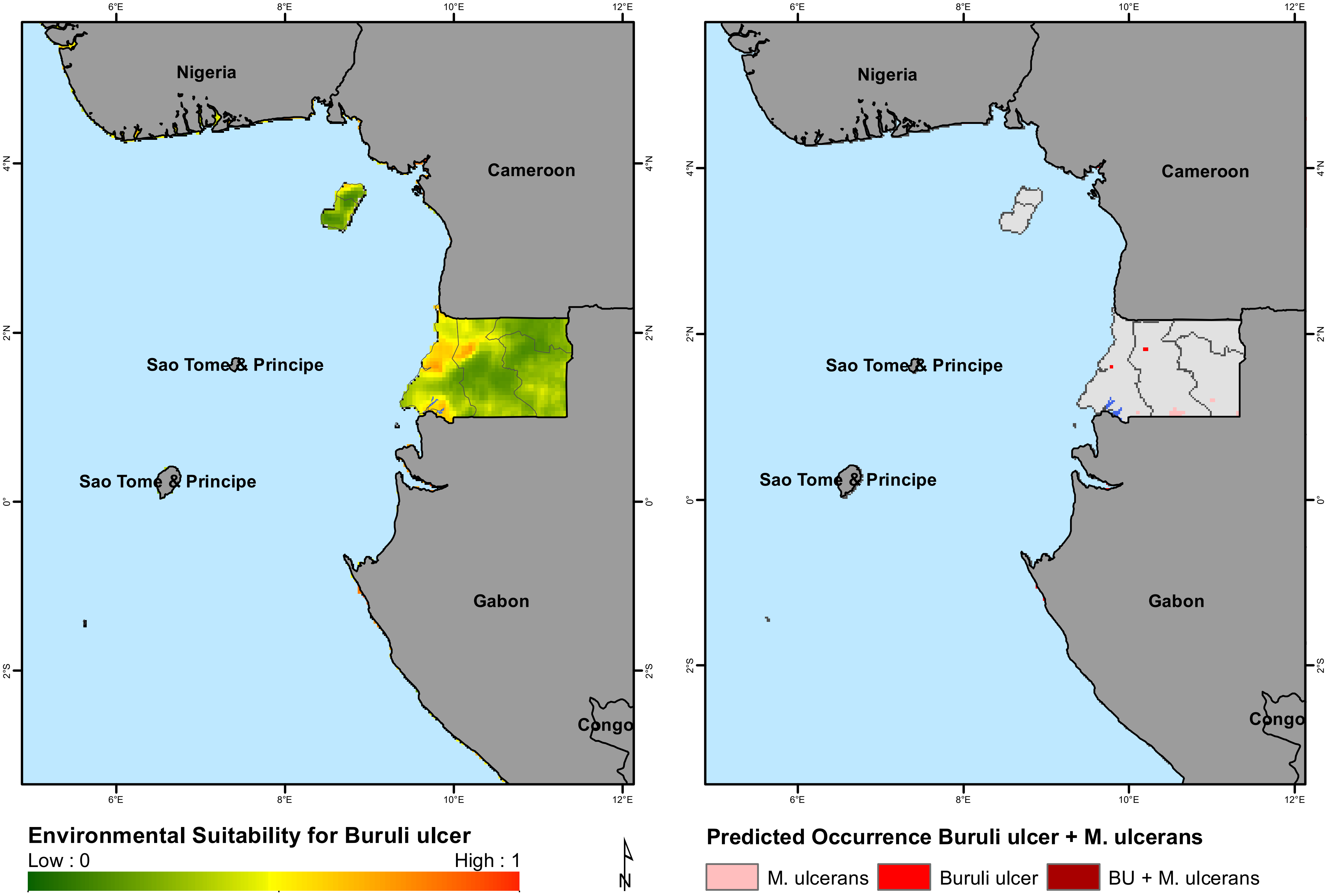

Gabon

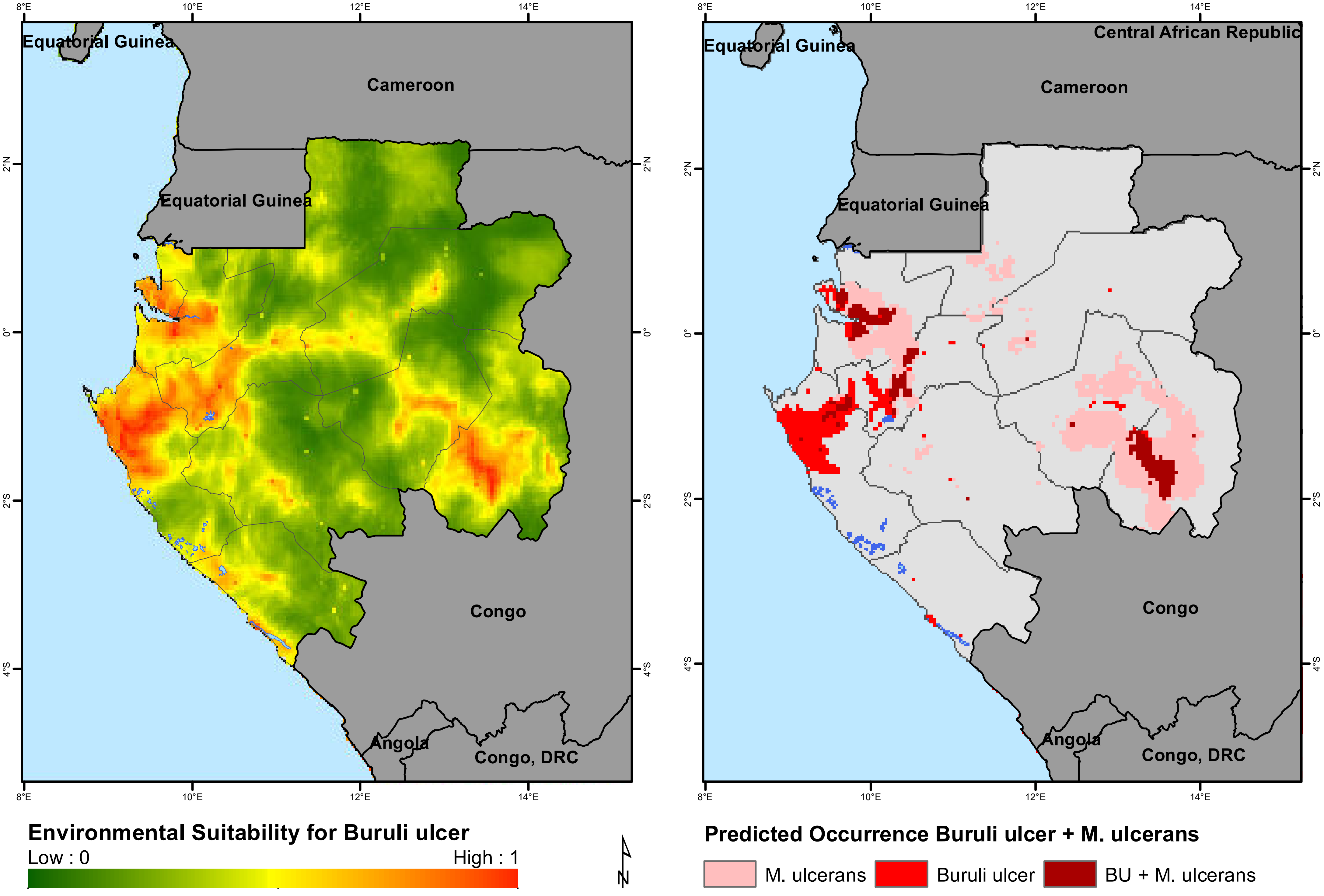

Equatorial Guinea
Cameroon
Equatorial Guinea
Congo
Angola
Congo, DRC
Central African Republic
8°E
10°E
12°E
14°E
2°N
0°
2°S
4°S
Environmental Suitability for Buruli ulcer
Low : 0
High : 1
N
Predicted Occurrence Buruli ulcer + M. ulcerans
M. ulcerans
Buruli ulcer
BU + M. ulcerans

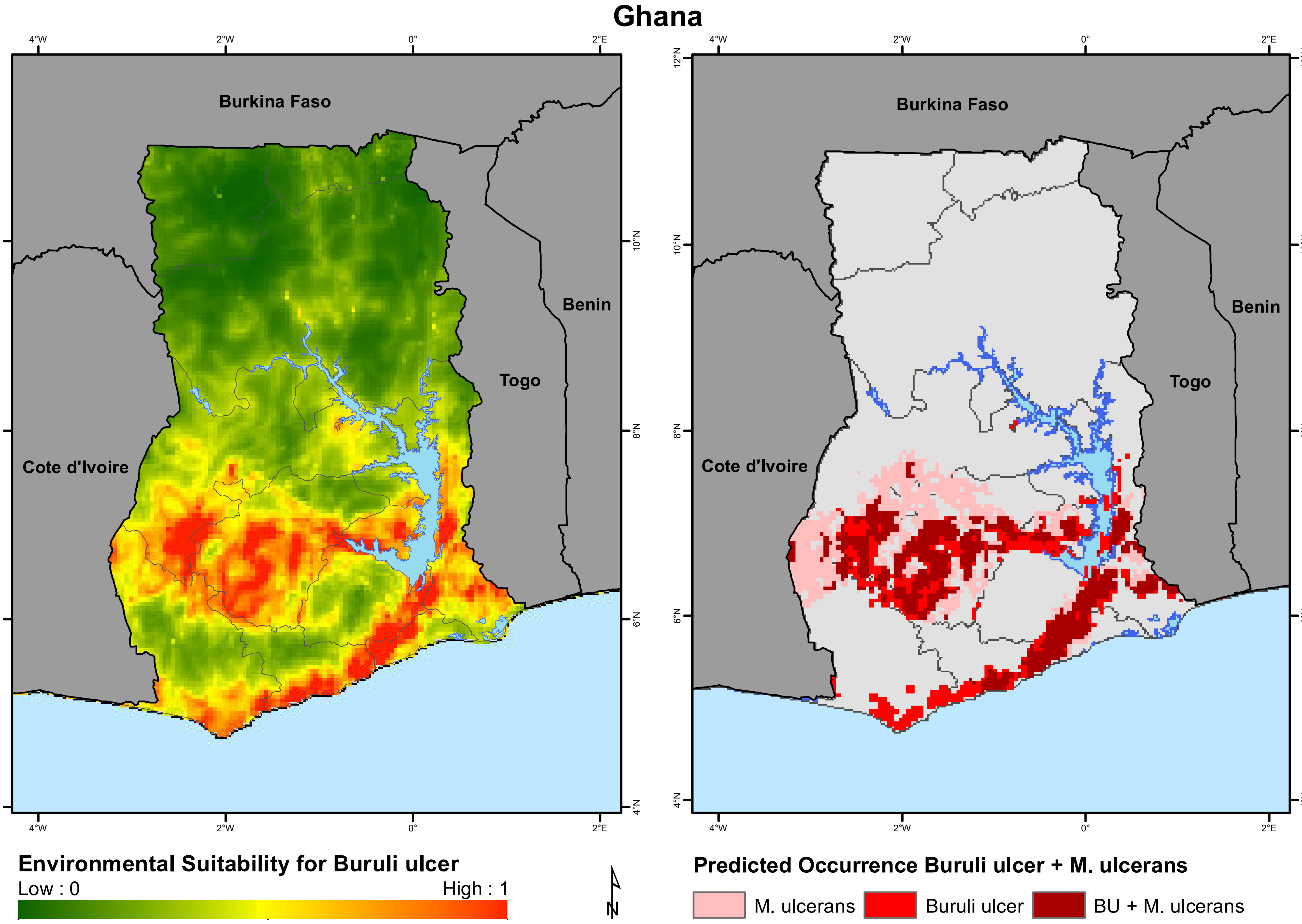
Ghana
Burkina Faso
Cote d'Ivoire
Togo
Benin
4°W
2°W
0°
2°E
4°N
6°N
8°N
10°N
12°N
Environmental Suitability for Buruli ulcer
Low : 0
High : 1
N
Predicted Occurrence Buruli ulcer + M. ulcerans
M. ulcerans
Buruli ulcer
BU + M. ulcerans

Guinea

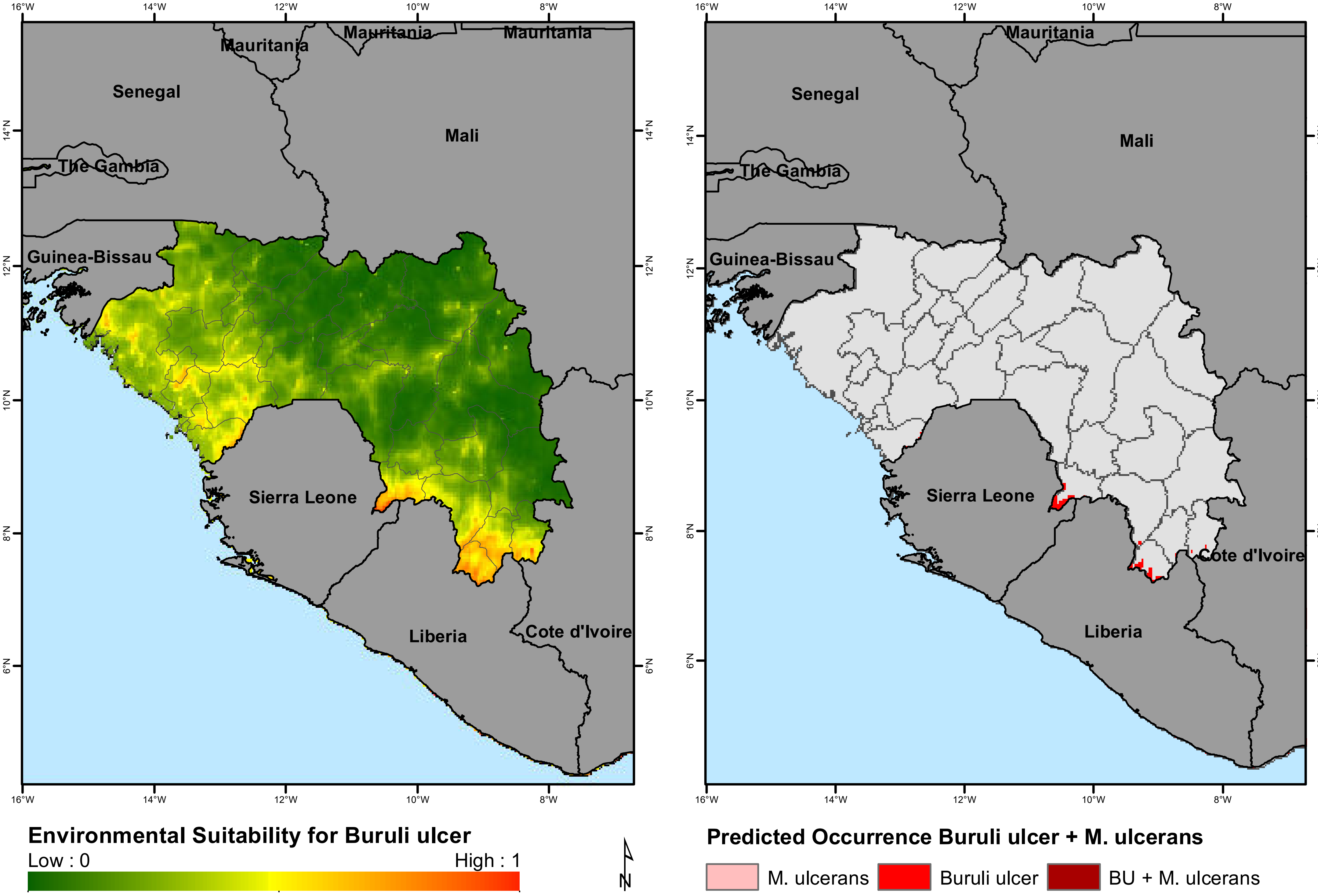
16°W
14°W
12°W
10°W
8°W
14°N
12°N
10°N
8°N
6°N
Mauritania
Senegal
Mali
The Gambia
Guinea-Bissau
Sierra Leone
Liberia
Cote d'Ivoire
Environmental Suitability for Buruli ulcer
Low : 0
High : 1
N
Predicted Occurrence Buruli ulcer + M. ulcerans
M. ulcerans
Buruli ulcer
BU + M. ulcerans

Kenya

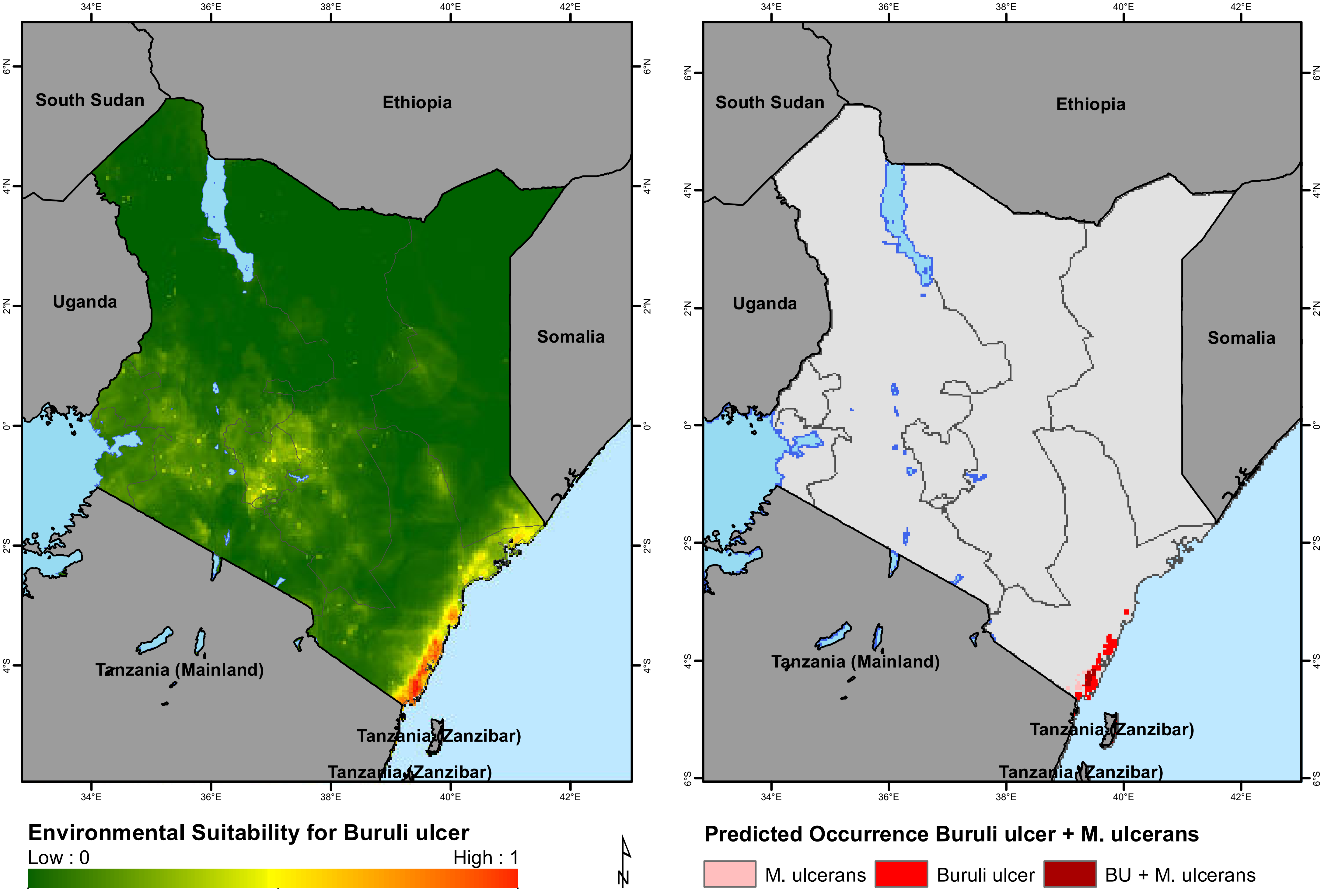

South Sudan
Ethiopia
Uganda
Somalia
Tanzania (Mainland)
Tanzania (Zanzibar)
Tanzania (Zanzibar)
34°E
36°E
38°E
40°E
42°E
6°N
4°N
2°N
0°
2°S
4°S
6°S
Environmental Suitability for Buruli ulcer
Low : 0
High : 1
N
Predicted Occurrence Buruli ulcer + M. ulcerans
M. ulcerans
Buruli ulcer
BU + M. ulcerans

Liberia

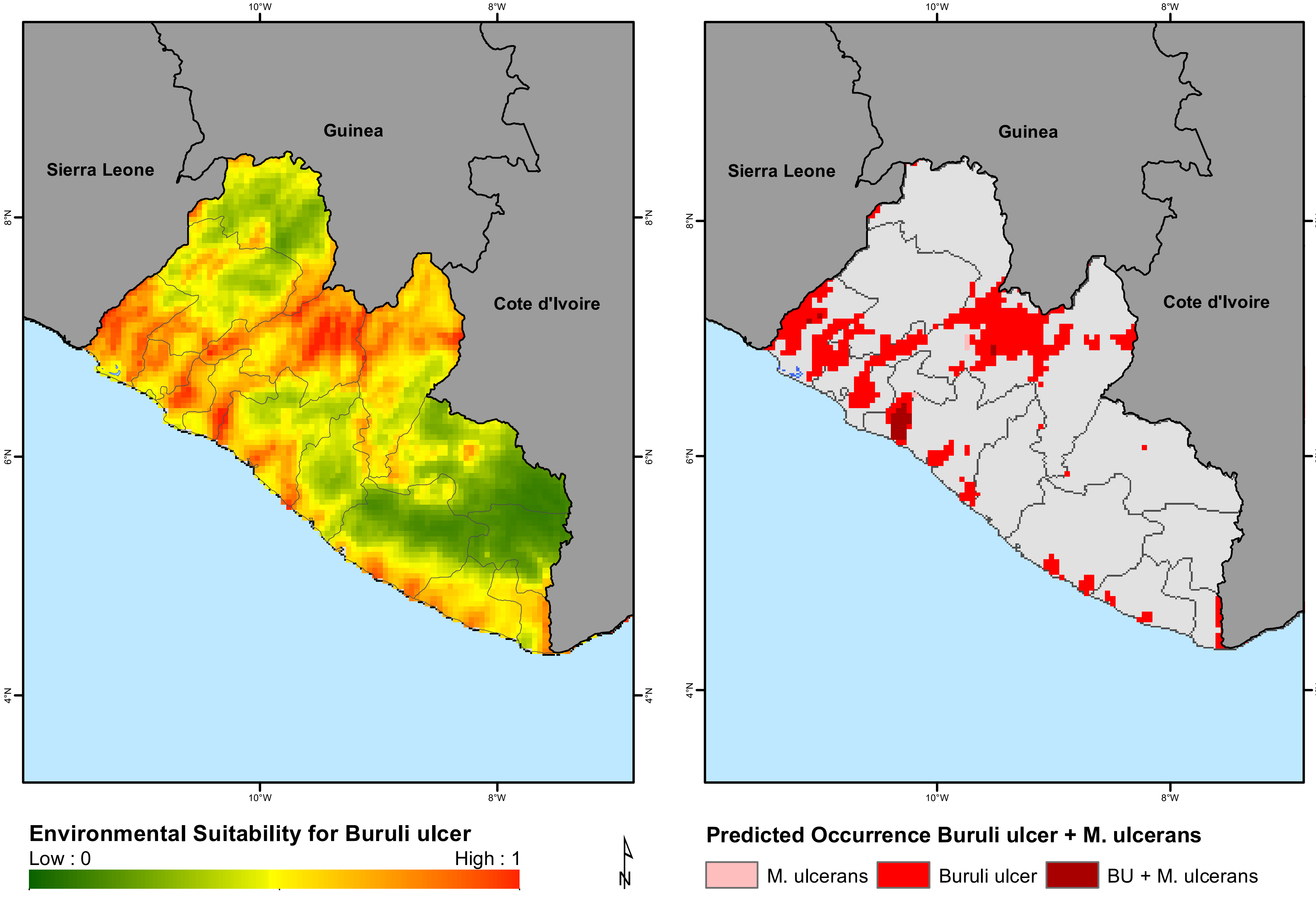

10°W
8°W
8°N
6°N
4°N
Sierra Leone
Guinea
Cote d'Ivoire
Environmental Suitability for Buruli ulcer
Low : 0
High : 1
N
Predicted Occurrence Buruli ulcer + M. ulcerans
M. ulcerans
Buruli ulcer
BU + M. ulcerans

Mozambique

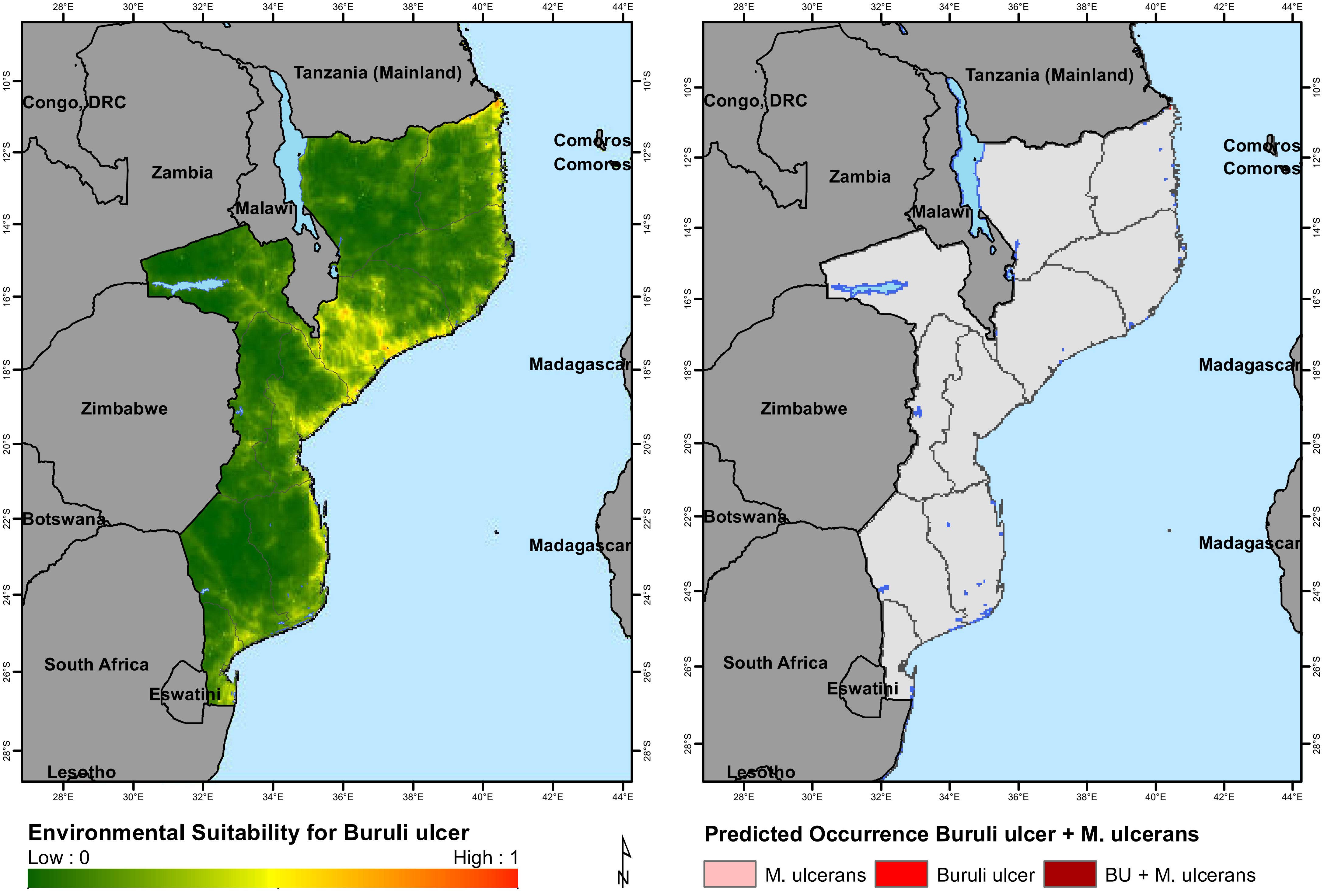
Tanzania (Mainland)
Congo, DRC
Zambia
Malawi
Comoros
Comoros
Madagascar
Madagascar
Zimbabwe
Botswana
South Africa
Eswatini
Lesotho
28°E
30°E
32°E
34°E
36°E
38°E
40°E
42°E
44°E
10°S
12°S
14°S
16°S
18°S
20°S
22°S
24°S
26°S
28°S
Environmental Suitability for Buruli ulcer
Low : 0
High : 1
N
Predicted Occurrence Buruli ulcer + M. ulcerans
M. ulcerans
Buruli ulcer
BU + M. ulcerans

Nigeria

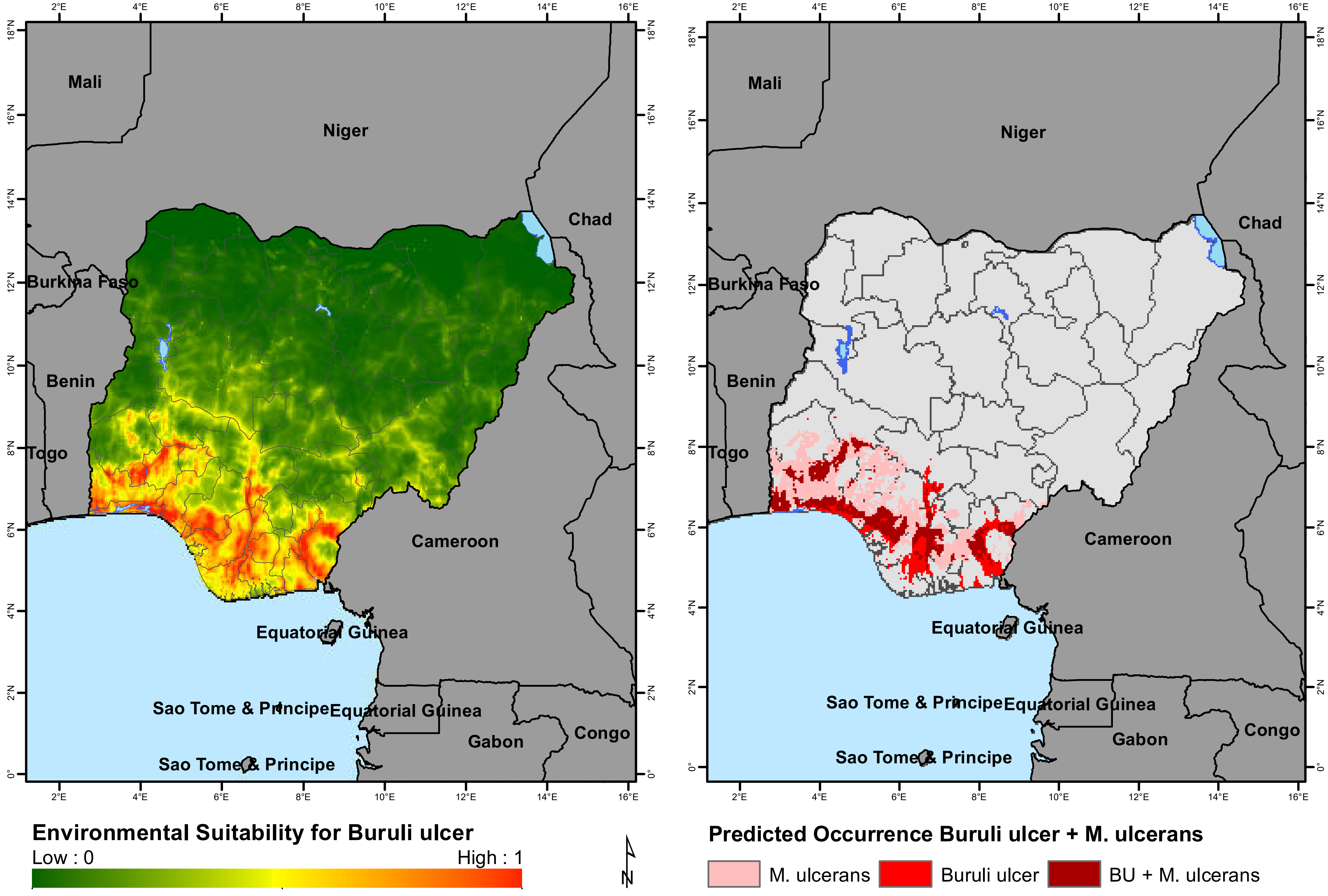
Mali
Niger
Chad
Burkina Faso
Benin
Togo
Cameroon
Equatorial Guinea
Sao Tome & Principe
Gabon
Congo
Environmental Suitability for Buruli ulcer
Low : 0
High : 1
N
Predicted Occurrence Buruli ulcer + M. ulcerans
M. ulcerans
Buruli ulcer
BU + M. ulcerans

Rwanda

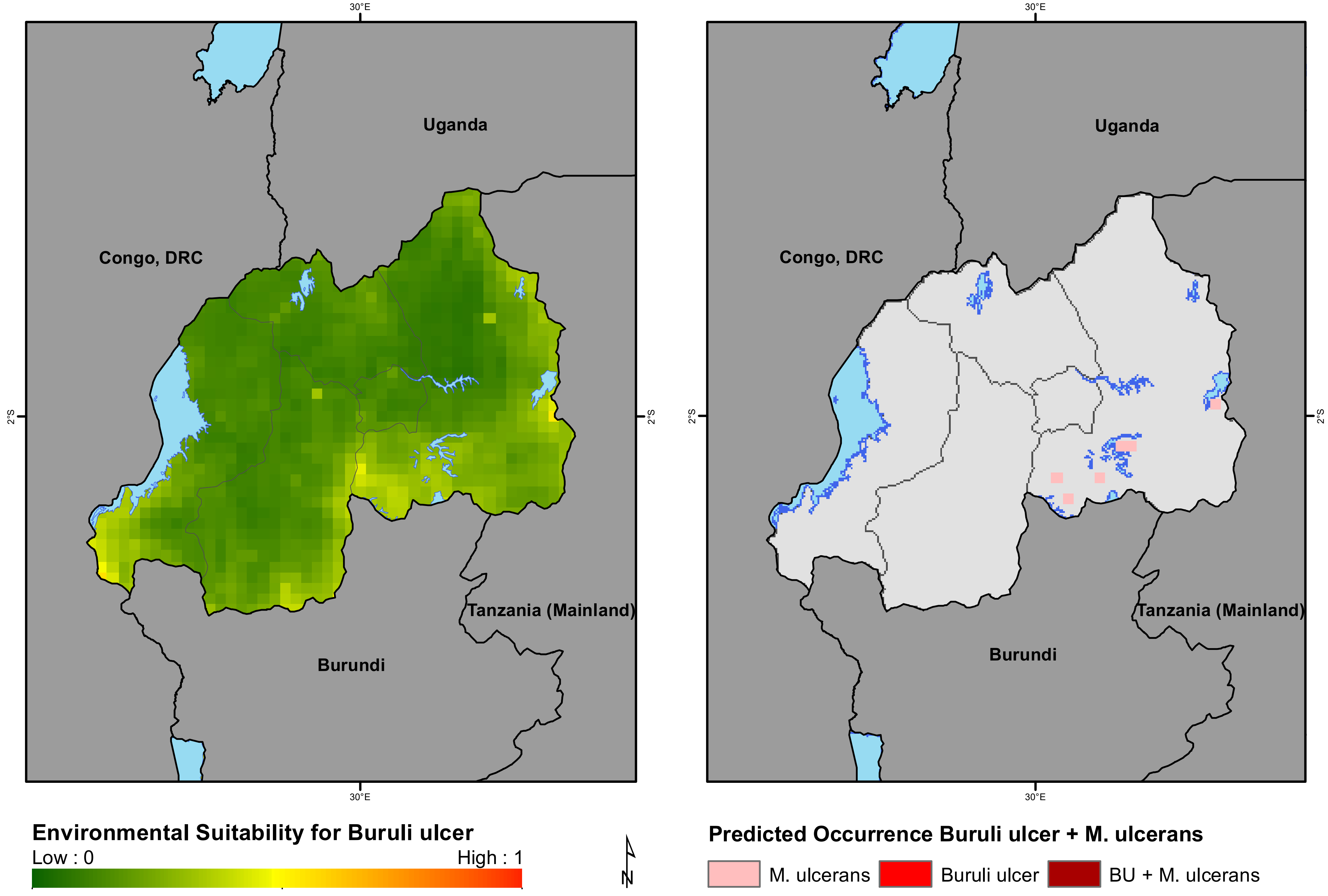
30°E
2°S
Uganda
Congo, DRC
Tanzania (Mainland)
Burundi
Environmental Suitability for Buruli ulcer
Low : 0
High : 1
N
Predicted Occurrence Buruli ulcer + M. ulcerans
M. ulcerans
Buruli ulcer
BU + M. ulcerans

Sierra Leone

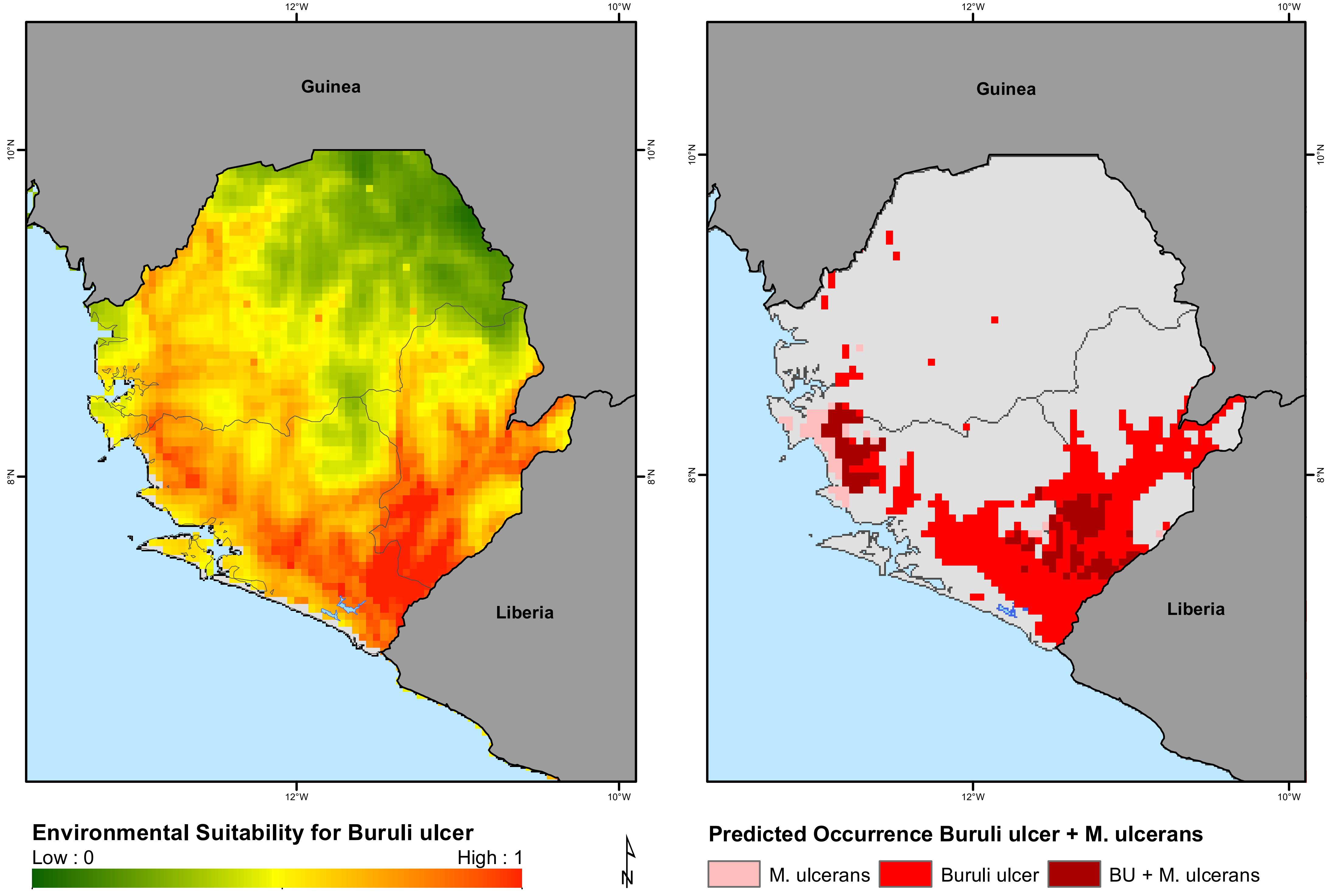

Tanzania (Mainland)

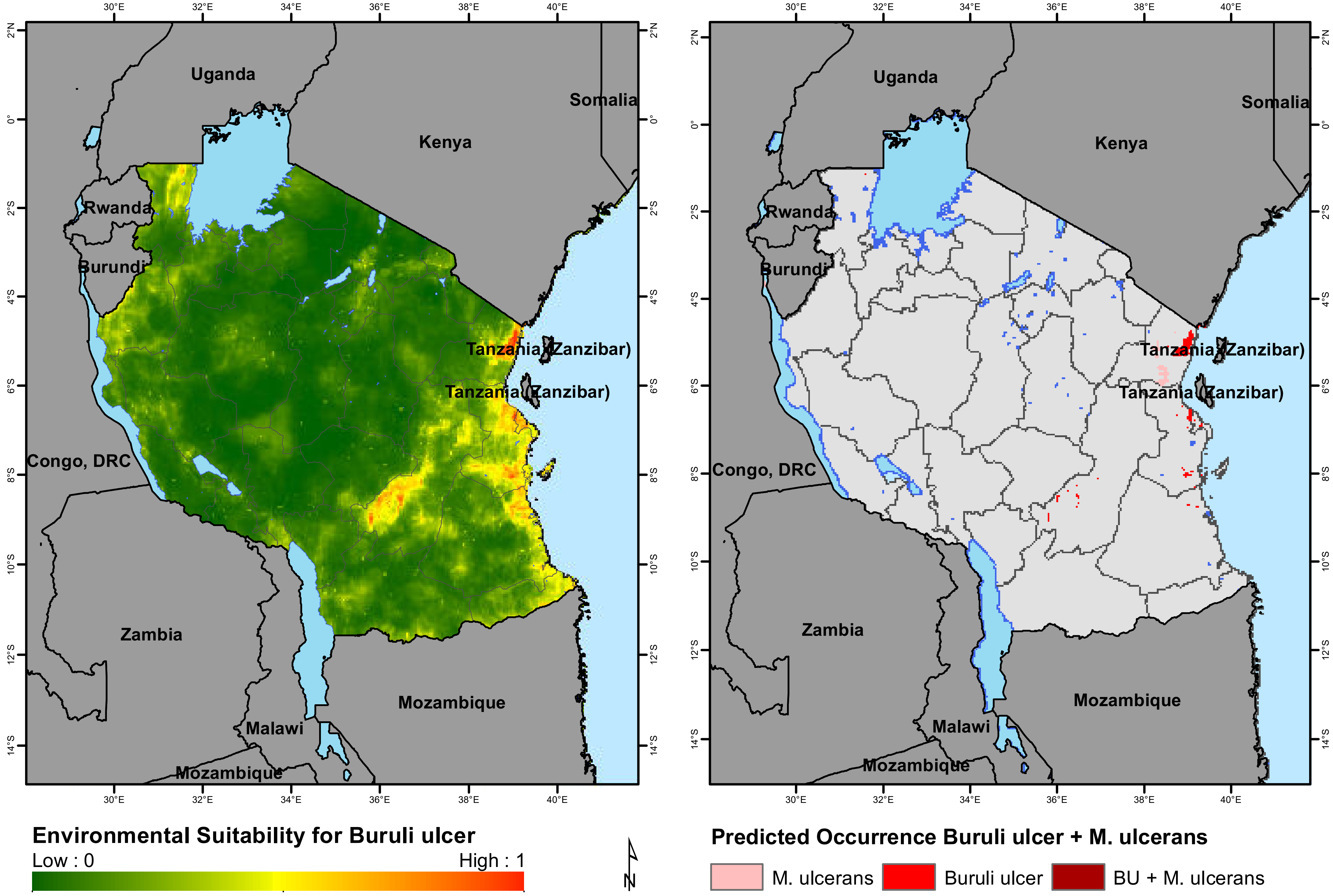

Togo

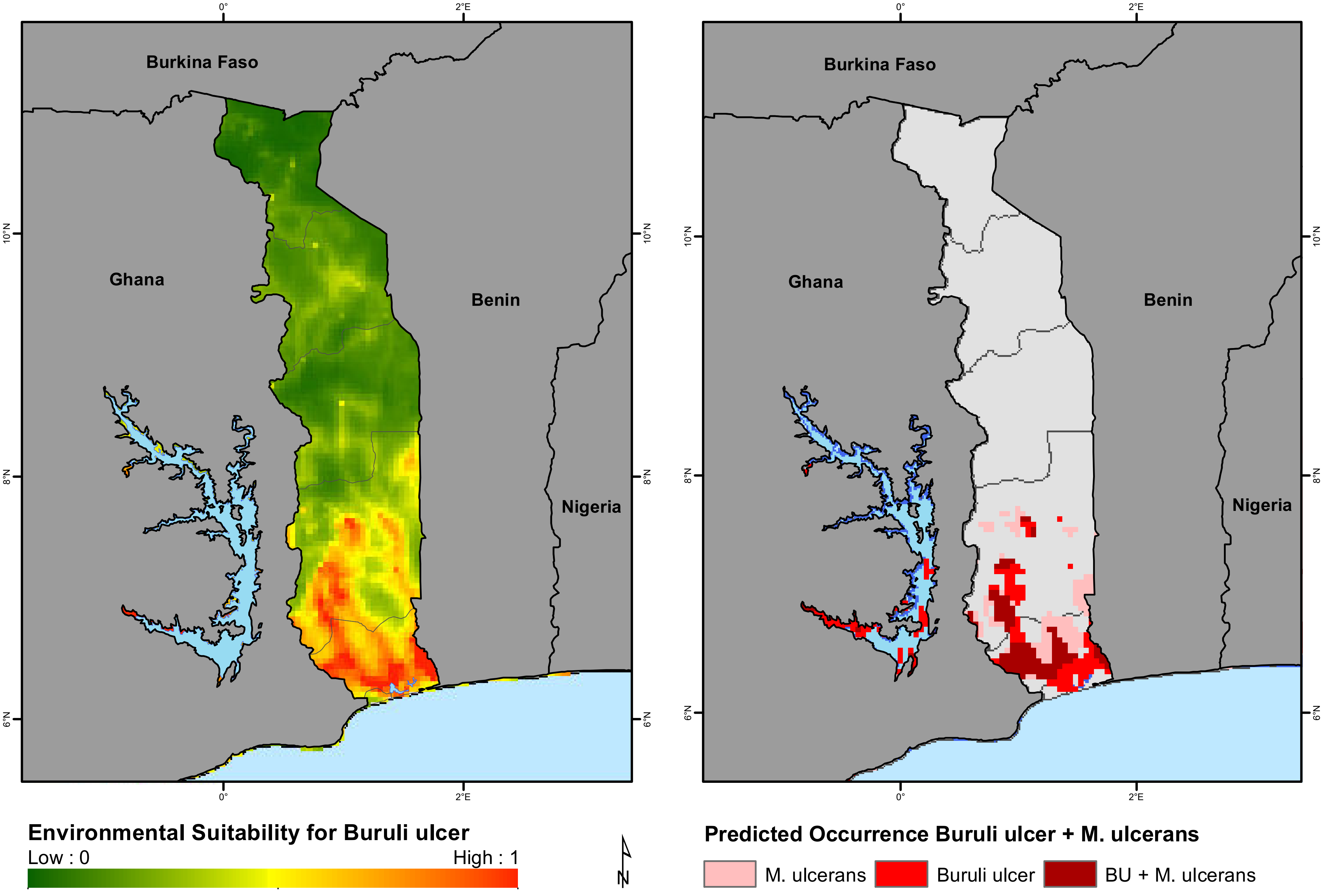
Burkina Faso
Ghana
Benin
Nigeria
0°
2°E
10°N
8°N
6°N
Environmental Suitability for Buruli ulcer
Low : 0
High : 1
N
Predicted Occurrence Buruli ulcer + M. ulcerans
M. ulcerans
Buruli ulcer
BU + M. ulcerans

Uganda

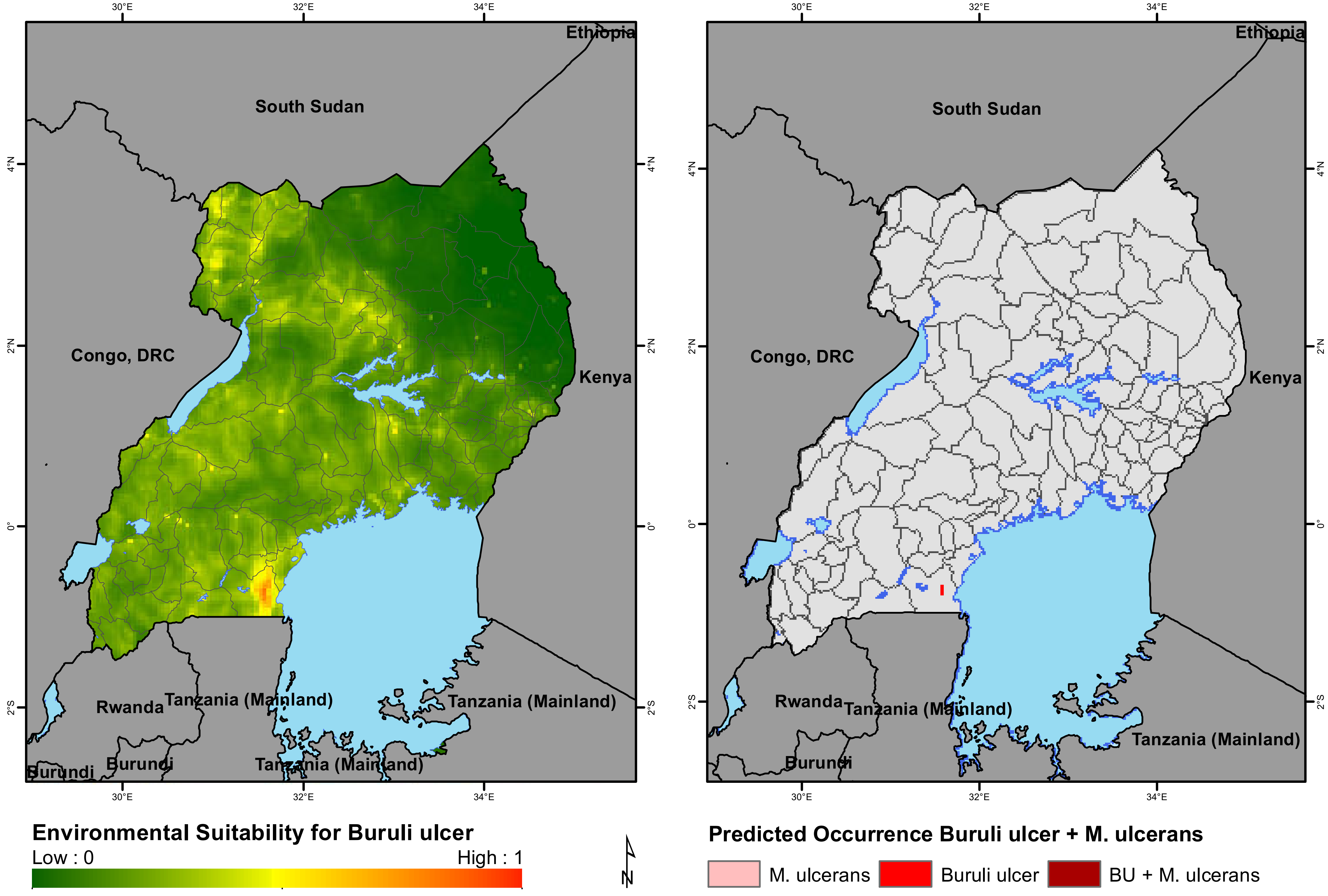
30°E
32°E
34°E
4°N
2°N
0°
2°S
Ethiopia
South Sudan
Congo, DRC
Kenya
Rwanda
Tanzania (Mainland)
Burundi
Environmental Suitability for Buruli ulcer
Low : 0
High : 1
N
Predicted Occurrence Buruli ulcer + M. ulcerans
M. ulcerans
Buruli ulcer
BU + M. ulcerans
