# Supplementary material for: Mapping suitability for Buruli ulcer at fine spatial scales across Africa: A modelling study
Source: PLoS Negl Trop Dis. 2021 Mar 3;15(3):e0009157. doi: 10.1371/journal.pntd.0009157 (PMC7959670; doi:10.1371/journal.pntd.0009157)
Supplement: S1 Text — (DOCX) [file pntd.0009157.s002.docx]

**S1 Text:** Selection of pseudoabsence and background points

Prior to building environmental models, we systematically generated pseudoabsence and background points which were integrated into the modelling framework as negative points.

Pseudoabsence points, intending to represent areas where BU or *M. ulcerans* were less likely to occur [1], were sampled from areas predicted to be unsuitable for the disease or bacterium, and at higher density from areas with lower evidence for BU occurrence. The area assumed to be unsuitable was delineated using the surface range envelope (SRE) function within the *biomod2* package, with the covariates selected as model predictors used as explanatory variables and the occurrence points (all locations) used as the response variable [2]. We used a threshold of 0.025 as the tolerance value so that the envelope represented the area within the 2.5^th^ and 97.5^th^ percentile of the range of each predictor variable [2]. The area outside of the SRE and within continental Africa was used as the extent for pseudoabsence selection. To weight the selection of pseudoabsence points according to the evidence of BU, we used the results of a previous literature review of BU endemicity [3]. The review quantified the strength of evidence for BU from multiple sources and assigned each country an evidence consensus score from -100 (consensus on absence of BU) to +100 (consensus of presence of BU). Upper sub-national administrative units with evidence of cases were scored from 0- 100 based on the contemporariness and diagnostic specificity of cases.

We rescaled national and sub-national evidence consensus scores to 0- 1 and linked both to upper administrative areas from the GADM [4] to generate an evidence consensus score layer. Sub-national areas with evidence of BU were assigned the sub-national evidence consensus score, and sub-national areas with no evidence of BU were assigned the national level score scaled by a factor of 0.5. The evidence consensus score layer was converted to a raster layer at 5x5km resolution representing the strength of evidence for BU.

Within the extent of pseudoabsence selection, we generated regular spatial points datasets at a scale of 5x5km. At each point we extracted the value of the evidence consensus raster (0- 1), and assigned a random score from 0- 1. Points with evidence consensus score lower than the randomly assigned value were defined as potential pseudoabsence points [5], resulting in a higher density of pseudoabsence points in areas of lower evidence consensus. Potential pseudoabsence points within 10km of occurrence points were excluded. Model pseudoabsences were selected at random from the potential pseudoabsences. Each pseudoabsence dataset contained the same number of points as the corresponding occurrence dataset. Pseudoabsences within the model of confirmed occurrences were selected from a random sub-sample of the human pseudoabsences representing all cases.

We additionally generated samples of background points, intended to account for the spatial bias of the occurrence points [6-8]. Gaussian kernel density surfaces, representing the density of occurrence points, were generated around recorded occurrence points with a bandwidth of 150km using the *Spatial kernel density estimate* function in the *spatialEco* package [8-10]. Samples of background points, each equal in size to its corresponding occurrence dataset, were selected from the density surfaces with probability defined by the kernel value (representing the smoothed density of occurrences at that location).

**References**

1. Barbet‐Massin M, Jiguet F, Albert CH, Thuiller W. Selecting pseudo‐absences for species distribution models: how, where and how many? Methods in ecology and evolution. 2012;3(2):327-38.

2. Wilfried Thuiller DG, Robin Engler and Frank Breiner. biomod2: Ensemble Platform for Species Distribution Modeling. R package version 3.3-15/r728. 2017.

3. Simpson H, Deribe K, Tabah EN, Peters A, Maman I, Frimpong M, et al. Mapping the global distribution of Buruli ulcer: a systematic review with evidence consensus. The Lancet Global Health. 2019;7(7):e912-e22.

4. Global Administrative Areas. GADM database of Global Administrative Areas, version 2.0 2012 [01/06/2018]. Available from: <http://www.gadm.org>

5. Bhatt S, Gething PW, Brady OJ, Messina JP, Farlow AW, Moyes CL, et al. The global distribution and burden of dengue. Nature. 2013;496(7446):504-7. doi: 10.1038/nature12060. PubMed PMID: 23563266; PubMed Central PMCID: PMCPMC3651993.

6. Fitzpatrick M, Gotelli N, Ellison A. MaxEnt versus MaxLike: empirical comparisons with ant species distributions. Ecosphere 4 (5): art 55. 2013.

7. Vollering J, Halvorsen R, Auestad I, Rydgren K. Bunching up the background betters bias in species distribution models. Ecography. 2019;42(10):1717-27.

8. Evans J. spatialEco. R package version 2.0-0. 2015.

9. Fourcade Y, Engler JO, Rödder D, Secondi J. Mapping species distributions with MAXENT using a geographically biased sample of presence data: a performance assessment of methods for correcting sampling bias. PloS one. 2014;9(5):e97122.

10. Evans J. spatialEco. 2019.
