# Supplementary material for: Mapping suitability for Buruli ulcer at fine spatial scales across Africa: A modelling study
Source: PLoS Negl Trop Dis. 2021 Mar 3;15(3):e0009157. doi: 10.1371/journal.pntd.0009157 (PMC7959670; doi:10.1371/journal.pntd.0009157)
Supplement: S2 Text — (DOCX) [file pntd.0009157.s003.docx]

**S2 Text:** Environmental variables used in modelling, including potential environmental predictors and their sources and the covariates that were included in the models of BU and *M. ulcerans* suitability.

Raster datasets of minimum and maximum temperature, precipitation in the wettest and driest quarters, and precipitation seasonality (representing the variation in monthly precipitation totals throughout the year) were obtained from the WorldClim v2.0 Global Climate Database [1].

Raster datasets representing annual potential evapo-transpiration (PET) and aridity, both derived from WorldClim datasets [1], were obtained from the Consortium for Spatial Information (CGIAR-CSI). PET quantifies atmospheric capacity to remove water from the air through evapotranspiration. The aridity index represents the balance of precipitation and atmospheric water demand [2]. An elevation dataset derived from data from the Shuttle Radar Topography Mission (SRTM) [3] was also obtained from CGIAR-CSI.

The topographic wetness index (TWI) raster was derived from the elevation raster as part of a previous modelling exercise [4]. The TWI represents the balance of flow accumulation (the potential to collect water) and drainage (the potential to lose water) of each cell, both based on the flow direction. Flow direction is the direction of steepest descent from each cell in the elevation dataset, calculated as: change in elevation value / distance * 100. Flow accumulation is derived by summing the flow direction value weights of all cells predicted to flow into each cell, and drainage is the sum of flow direction value weights of cells predicted to flow from each cell. Using these intermediary datasets, the TWI was generated using the algorithm

*TWI*=ln(*a/tanβ*)

where *a* is the Specific Catchment Area (SCA) for each cell, obtained from the flow accumulation layer, and *β* is the local slope around the cell, quantifying the potential for drainage.

Waterbodies and waterways were downloaded from the OpenStreetMap project (OSM) [5] through the platform *Geofabrik* [6]. Two separate datasets, one of rivers and streams and one of dams, were extracted from OpenStreetMap.

Raster surfaces showing tree-covered and intact forest at 250m resolution in 2015 were obtained from the Open Land Data service (LandGIS) [7,8]. These datasets were generated using data from the UNEP historic forest cover map [9], the ESA time series of land cover maps 2000–2015 [10] and data on intact forest landscape for 2000, 2013 and 2016 [11].

Land cover datasets obtained from the European Space Agency’s Landcover project [12] were used to define areas of agricultural land (crops).

We used spatial analyst tools in ArcGIS 10.3 software (ESRI Inc., Redlands CA, USA) to generate continuous surfaces of straight line (Euclidean) distance to waterbodies; waterways; dams; rivers and streams; deforested areas; and agricultural land, at a spatial resolution of 5km x 5km.

A raster dataset of long-term averaged Enhanced Vegetation Index (EVI) from 2000-2015 was calculated from yearly EVI estimates obtained from the Vegetation Index and Phenology (VIPPHEN) global datasets [13]. The gridded continuous VIPPHEN data products, provided globally at 0.05-degree spatial resolution, were downloaded from the Earth Explorer NASA site (<https://earthexplorer.usgs.gov/>). The EVI quantifies vegetation cover based on the relative levels of different wavelengths of radiation detected by the MODIS satellite, operated by the National Aeronautics and Space Administration (NASA) [13]. The EVI was selected over other available vegetation indices as it retains higher sensitivity in densely vegetated areas, and is more robust to interference from the canopy background signal than other vegetation indices [13].

**Table A**: Potential environmental predictors and their sources, indicating the covariates that were included in the models of Buruli ulcer and *M. ulcerans* suitability.

| **Variable** | **Included in model** | | **Source** |
| --- | --- | --- | --- |
|  | **BU** | **MU** |  |
| **Precipitation of Driest Month** | ✓ | ✓ | WorldClim v2.0 Global Climate Database [1] |
| **Precipitation Seasonality** | ✓ |  |  |
| **Precipitation of Wettest Quarter** | ✓ |  |  |
| **Minimum temperature** | ✓ | ✓ |  |
| **Maximum temperature** |  |  |  |
| **Annual potential evapotranspiration** | ✓ | ✓ | CGIAR-CSI [14] |
| **Aridity index** |  |  |  |
| **Elevation** |  |  |  |
| **Topographic wetness index*** |  | ✓ | Derived from elevation [4] |
| **Distance to waterbodies** | ✓ | ✓ | Derived from data from Open Street Map [5] |
| **Distance to rivers and streams** | ✓ | ✓ |  |
| **Distance to dams** | ✓ | ✓ |  |
| **Distance to deforested areas** | ✓ | ✓ | Derived from data from LandGIS [8] |
| **Distance to agricultural land** | ✓ | ✓ | Derived from Global Land Cover 2000 [15] |

BU = Buruli ulcer, MU = *M. ulcerans*
* Topographic wetness index was selected for the BU suitability model but was dropped after the initial modelling step as it made little contribution to the model.

**References**

1. WorldClim. Global Climate data.

2. Zomer RJ, Trabucco A, Bossio DA, Verchot LV. Climate change mitigation: A spatial analysis of global land suitability for clean development mechanism afforestation and reforestation. Agriculture, ecosystems & environment. 2008;126(1-2):67-80.

3. Jarvis A, H.I. Reuter, A. Nelson, E. Guevara. Hole-filled SRTM for the globe Version 4, available from the CGIAR-CSI SRTM 90m Database 2008 [27/01/2017]. Available from: <http://srtm.csi.cgiar.org>.

4. Cano J, Rodriguez A, Simpson H, Tabah EN, Gomez JF, Pullan RL. Modelling the spatial distribution of aquatic insects (Order Hemiptera) potentially involved in the transmission of Mycobacterium ulcerans in Africa. Parasit Vectors. 2018;11(1):501. doi: 10.1186/s13071-018-3066-3. PubMed PMID: 30189883.

5. OpenStreetMap contributors. 2015 [16/04/2019]. Available from: <https://planet.openstreetmap.org>.

6. Jochen Topf and Frederik Ramm. Geofabrik 2007. Available from: [www.geofabrik.de](http://www.geofabrik.de).

7. Hengl T. Tree-covered and intact forest landscapes BC1000, 1995, 2000, 2005, 2010, 2013, 2016 at 250 m. 2018.

8. Hengl TaK, M. LandGIS — Open Land Data service. Available from: <https://github.com/Envirometrix/LandGISmaps#landgis--open-land-data-service>.

9. Generalised Original and Current Forest. In: Centre UEWCM, editor. 1998.

10. Agency ES. [04/01/2019]. Available from: <http://www.esa.int/ESA>.

11. Potapov P. YA, Turubanova S., Dubinin M., Laestadius L., Thies C., Aksenov D., Egorov A., Yesipova Y., Glushkov I., Karpachevskiy M., Kostikova A., Manisha A., Tsybikova E., Zhuravleva I. Intact forest landscape (IFL 2000, 2013 and 2016). 2013.

12. Team ESACCIL. Land cover maps for 2000, 2005 and 2010 2014 [04/01/2019]. Available from: <https://www.esa-landcover-cci.org/>.

13. Didan K. MOD13Q1 MODIS/Terra Vegetation Indices 16-Day L3 Global 250m SIN Grid V006. 2015.

14. CGIAR-CSI: Consortium for Spatial Information [27/01/2017]. Available from: <http://www.cgiar-csi.org>.

15. service JRCTECssak. Global Land Cover 2000 - Products [15/02/2019]. Available from: <https://forobs.jrc.ec.europa.eu/products/glc2000/products.php>.
