# Supplementary figures and images for: Mapping suitability for Buruli ulcer at fine spatial scales across Africa: A modelling study
Source: PLoS Negl Trop Dis. 2021 Mar 3;15(3):e0009157. doi: 10.1371/journal.pntd.0009157 (PMC7959670; doi:10.1371/journal.pntd.0009157)

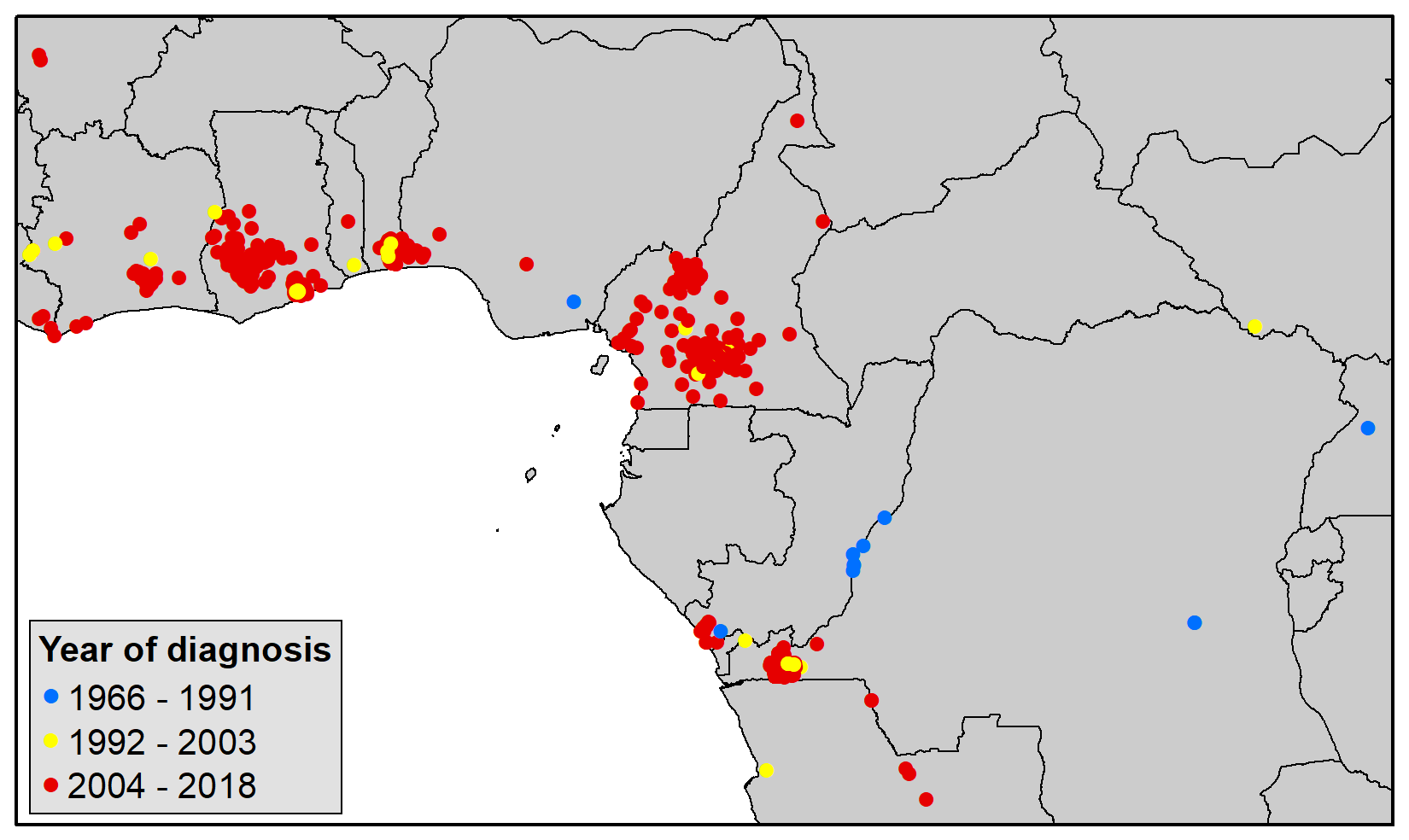

Supplement: S1 Fig — (TIF) [file pntd.0009157.s004.tif]

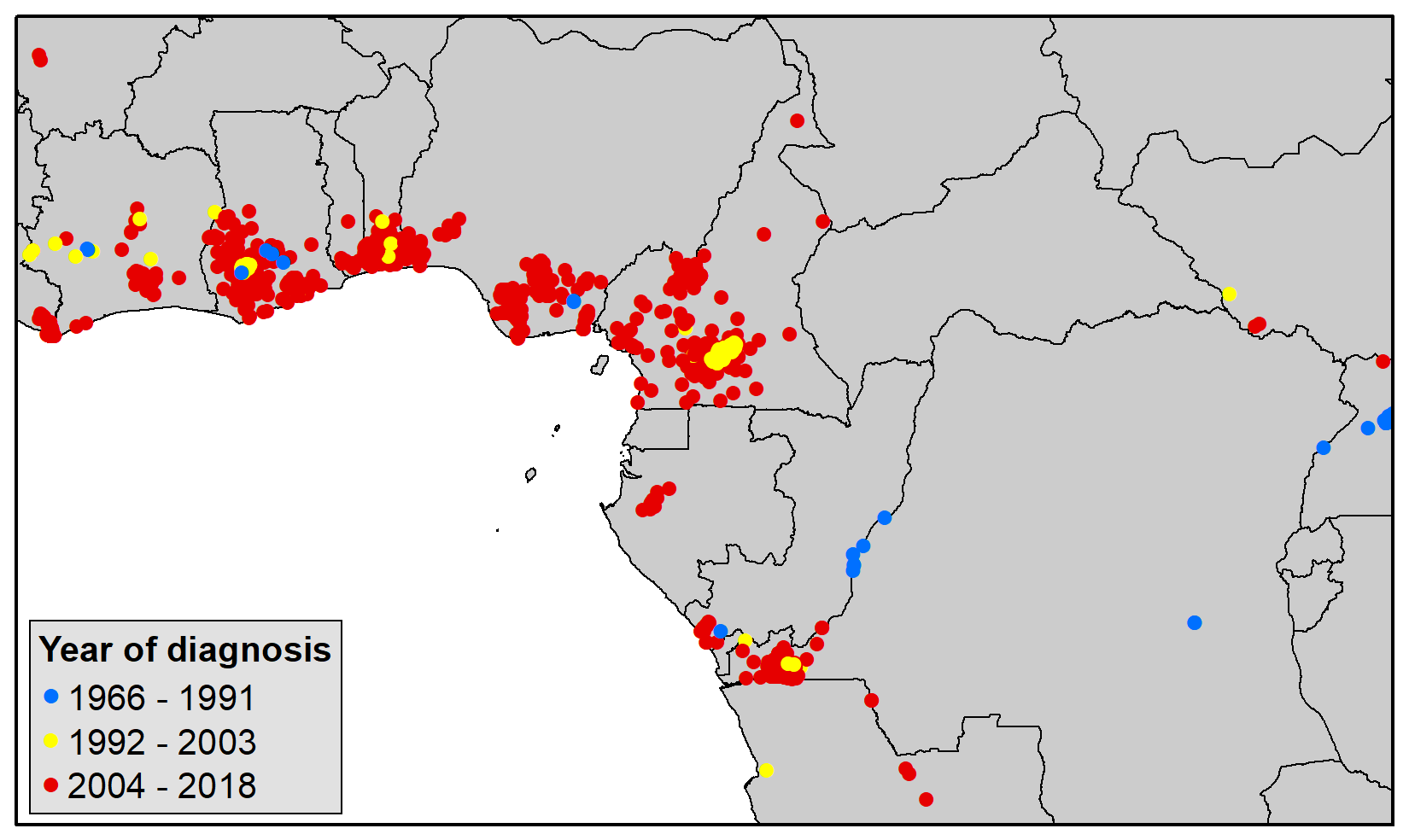

Supplement: S2 Fig — (TIF) [file pntd.0009157.s005.tif]

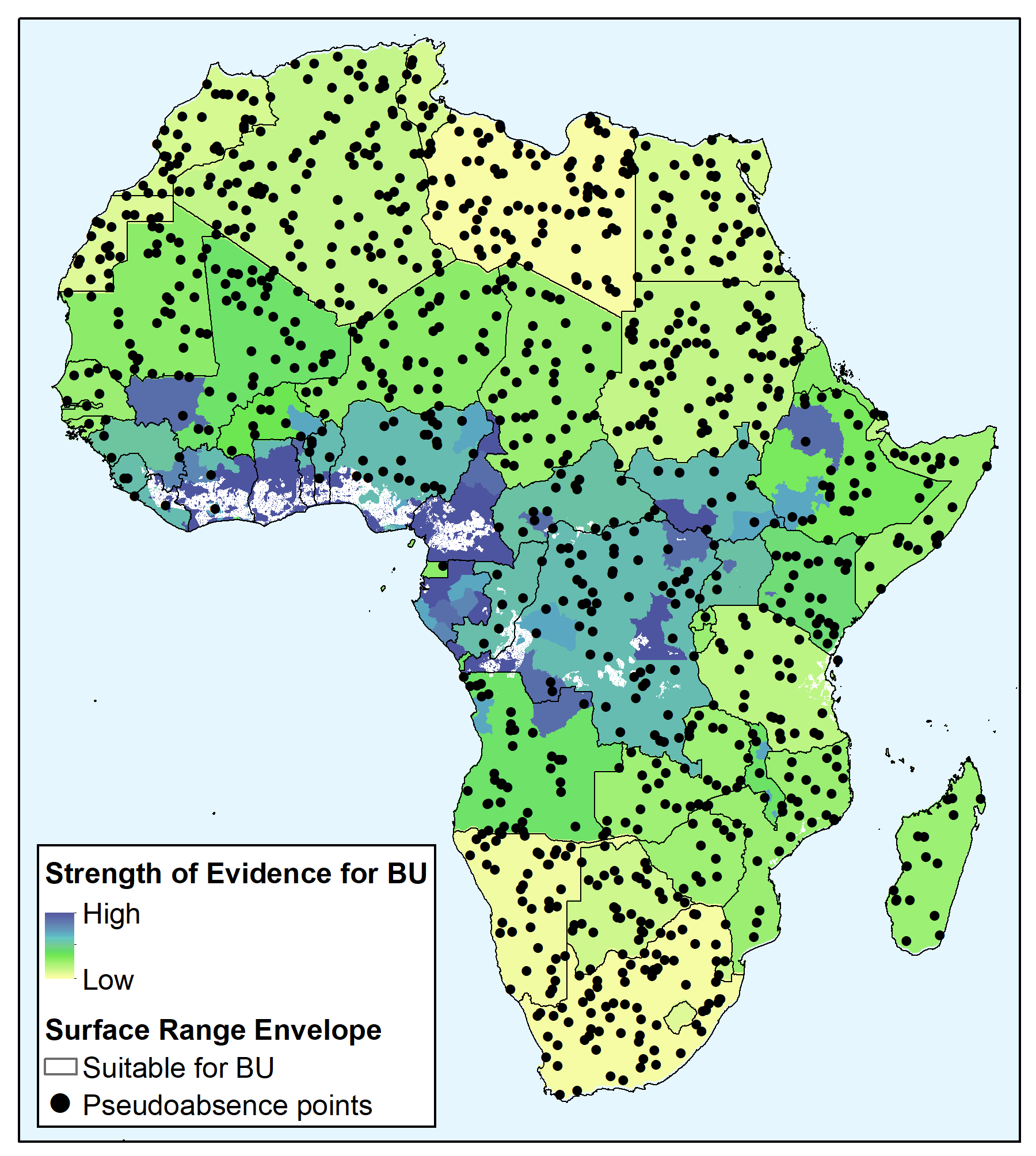

Supplement: S3 Fig — Pseudoabsence points were selected outside of the BU surface range envelope (white; the area containing values between the 2.5th and 97.5th percentile of all predictor variables) and selection was biased according to the strength of evidence for BU at national or subnational level (yellow to blue shading) using results from Simpson et al. Lancet Glob. Health 2019. (TIF) [file pntd.0009157.s006.tif]

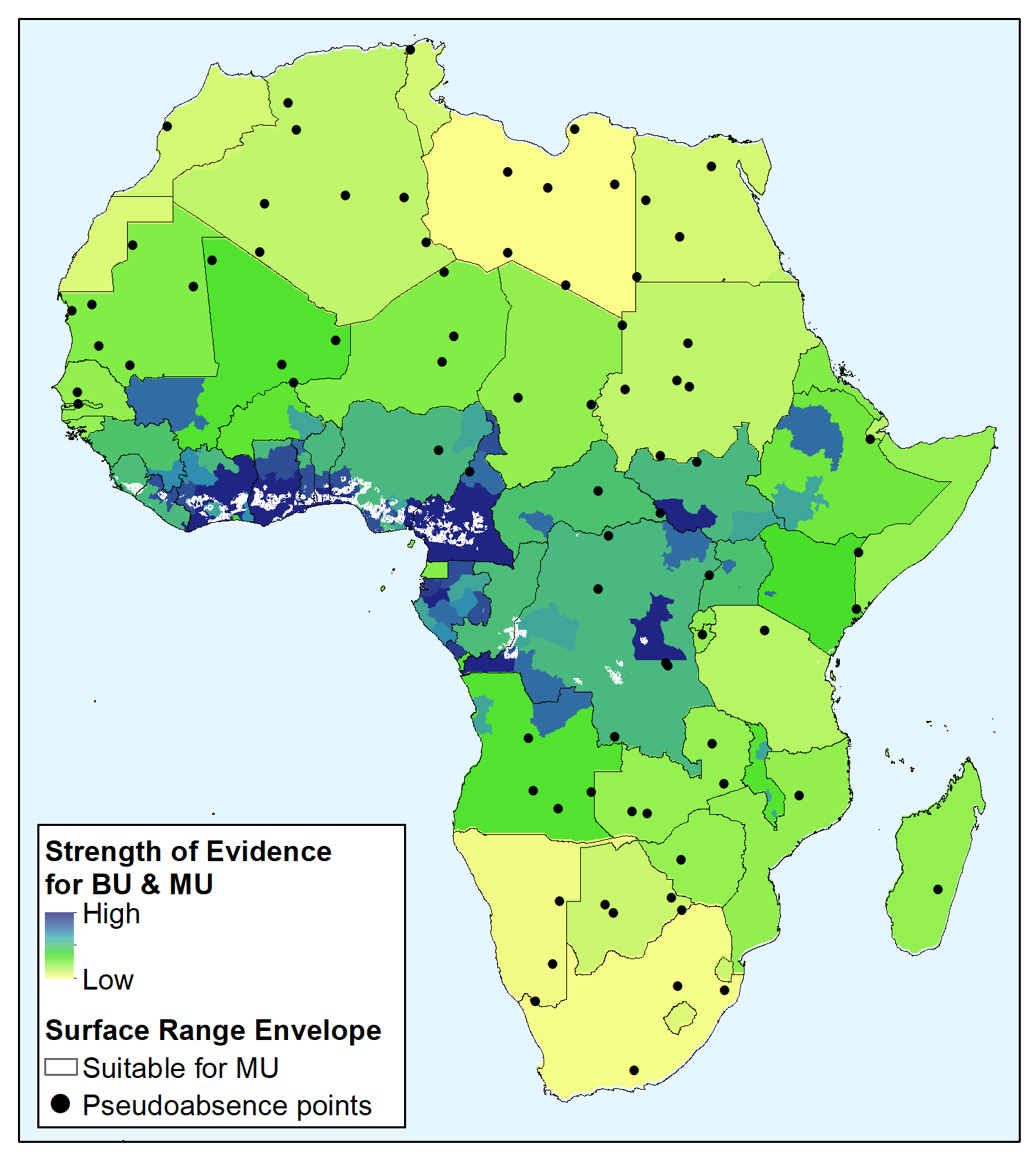

Supplement: S4 Fig — Pseudoabsence points were selected outside of the MU surface range envelope (white; the area containing values between the 2.5th and 97.5th percentile of all predictor variables) and selection was biased according to the strength of evidence for BU and MU at national or subnational level (yellow to blue shading) using results from Simpson et al. Lancet Glob. Health 2019. (TIF) [file pntd.0009157.s007.tif]

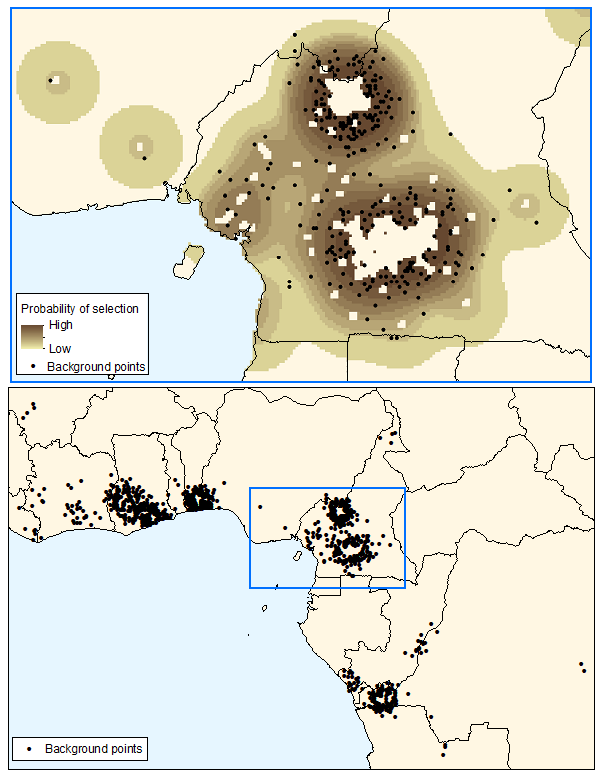

Supplement: S5 Fig — Background points were restricted to a minimum distance of 10km from human occurrence points (not shown on the map) and were selected with probability defined by the kernel density surface representing the density of occurrence points. (TIF) [file pntd.0009157.s008.tif]

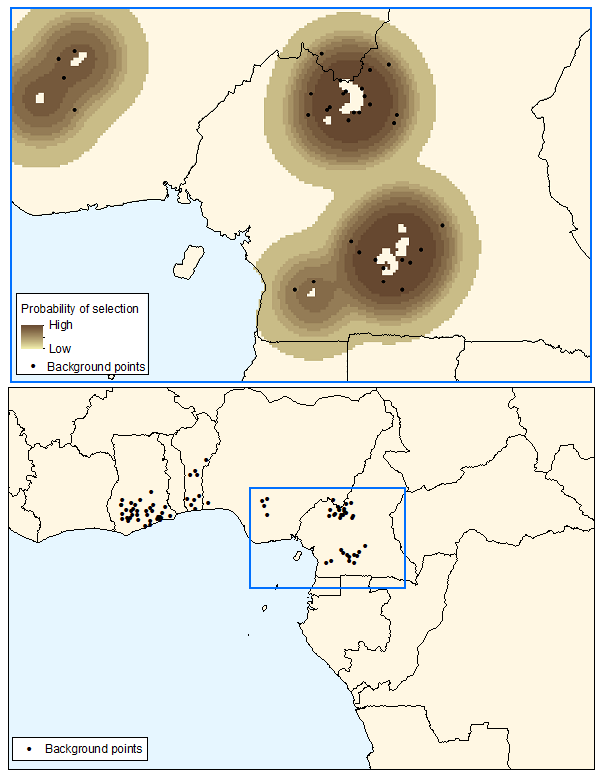

Supplement: S6 Fig — Background points were restricted to a minimum distance of 10km from human or environmental occurrence points (not shown on the map) and were selected with probability defined by the kernel density surface representing the density of occurrence points. (TIF) [file pntd.0009157.s009.tif]

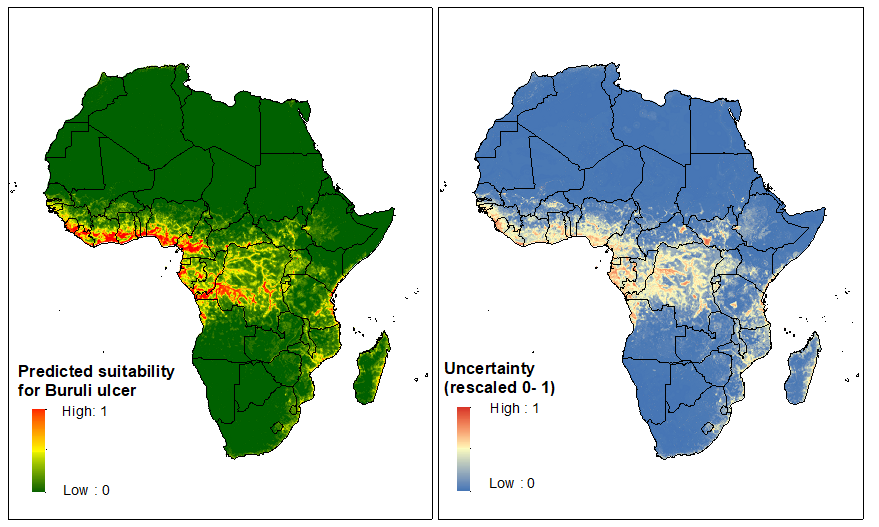

Supplement: S7 Fig — (TIF) [file pntd.0009157.s010.tif]

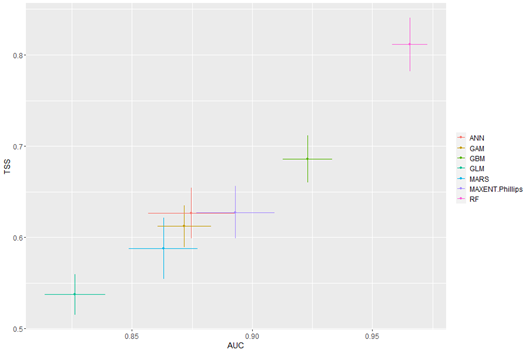

Supplement: S8 Fig — Performance evaluated in terms of the mean true skill statistic (TSS) and the mean area under the curve (AUC) of the receiver operation characteristic. (TIF) [file pntd.0009157.s011.tif]

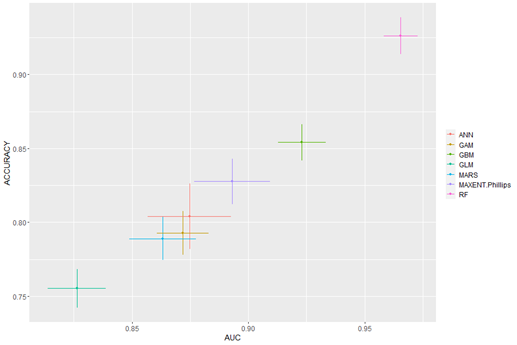

Supplement: S9 Fig — Performance evaluated in terms of accuracy (percent correctly classified) and the mean area under the curve (AUC) of the receiver operation characteristic. Individual model algorithms: ANN = artificial neural networks; GAM = generalized additive models; GBM = generalized boosted regression models; GLM = generalized linear models; MARS = multiple adaptive regression splines; MAXENT. Phillips = maximum entropy; RF = random forest. (TIF) [file pntd.0009157.s012.tif]

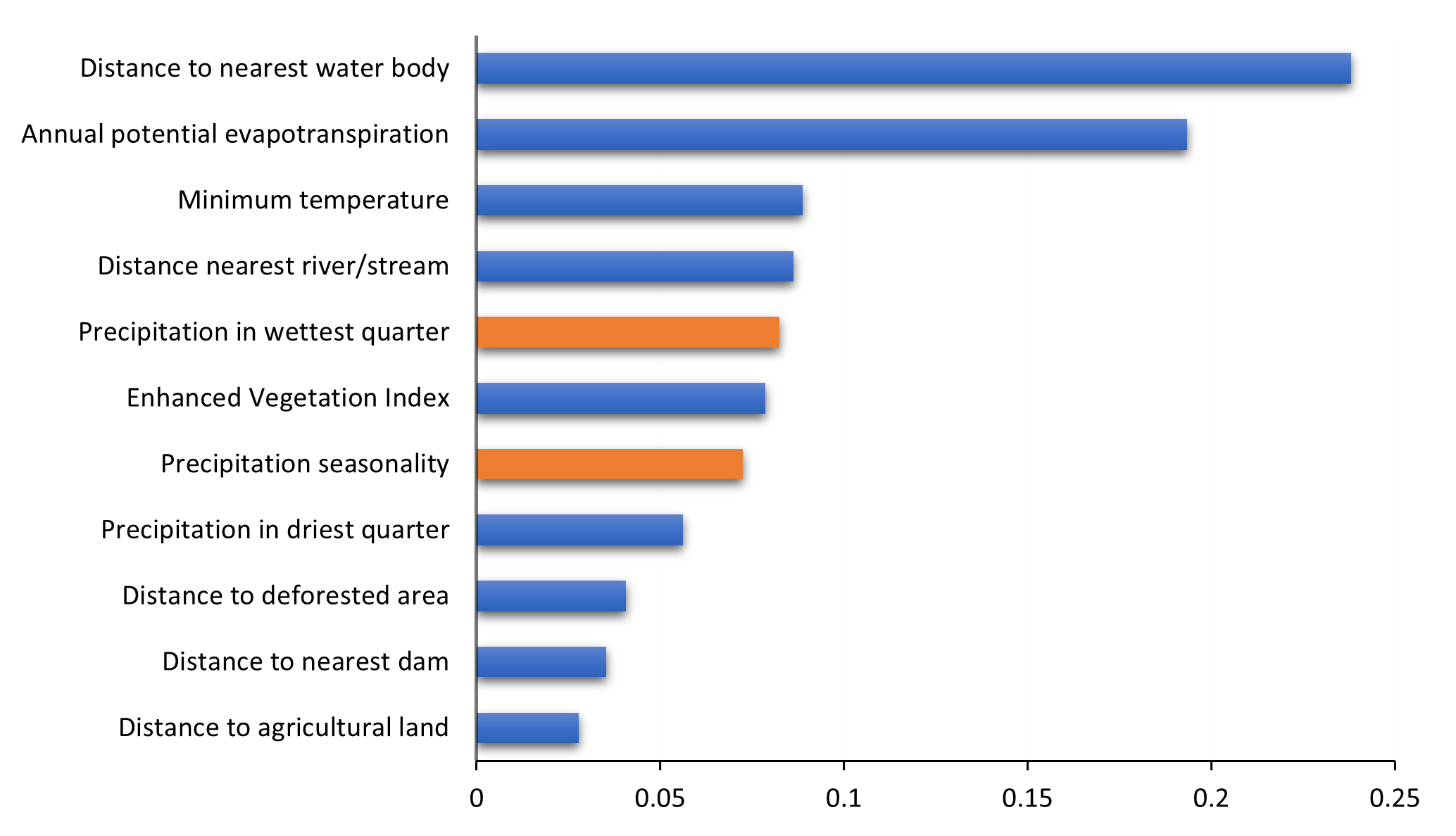

Supplement: S10 Fig — Shows contribution of variables to model for Buruli ulcer. Blue bars = variables selected as predictors of BU occurrence and M. ulcerans in the environment. Orange bars = variables selected as predictors of Buruli ulcer (BU) occurrence only. (TIF) [file pntd.0009157.s013.tif]

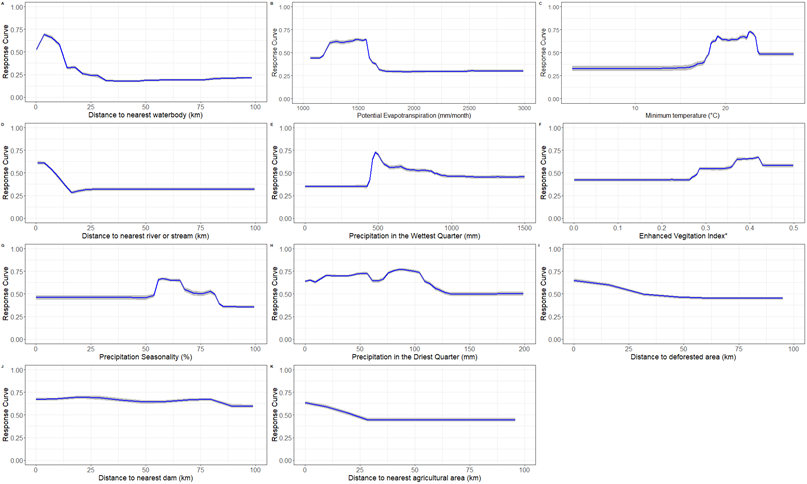

Supplement: S11 Fig — Marginal Effect of Environmental Predictors on Environmental Suitability for Buruli ulcer (TIF) [file pntd.0009157.s014.tif]

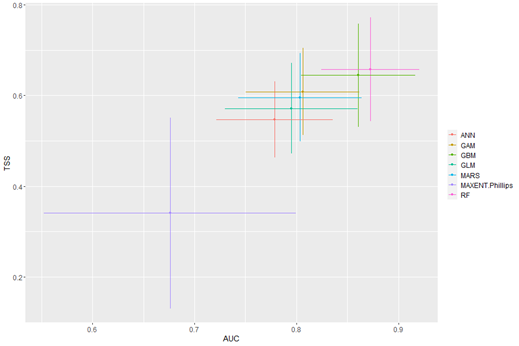

Supplement: S12 Fig — Performance evaluated in terms of the mean true skill statistic (TSS) and the mean area under the curve (AUC) of the receiver operation characteristic. (TIF) [file pntd.0009157.s015.tif]

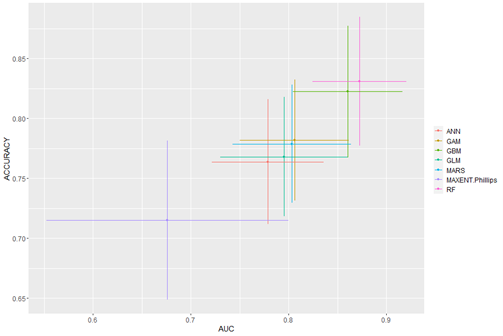

Supplement: S13 Fig — Performance evaluated in terms of accuracy (percent correctly classified) and the mean area under the curve (AUC) of the receiver operation characteristic. Individual model algorithms: ANN = artificial neural networks; GAM = generalized additive models; GBM = generalized boosted regression models; GLM = generalized linear models; MARS = multiple adaptive regression splines; MAXENT. Phillips = maximum entropy; RF = random forest. (TIF) [file pntd.0009157.s016.tif]

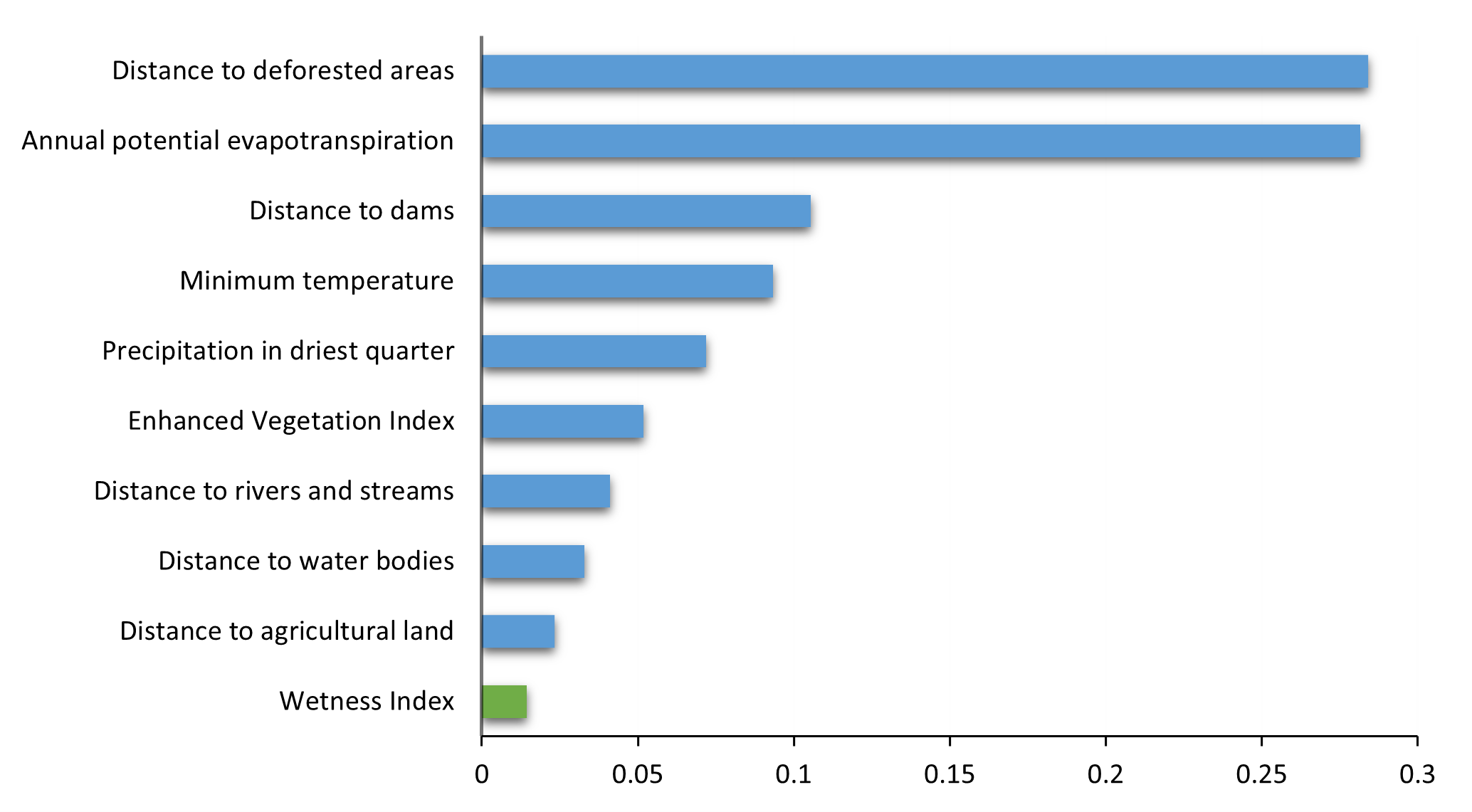

Supplement: S14 Fig — Shows contribution of variables to model for Mycobacterium ulcerans. Blue bars = variables selected as predictors of BU occurrence and M. ulcerans in the environment Green bars = variables selected as predictors of M. ulcerans in the environment only (TIF) [file pntd.0009157.s017.tif]

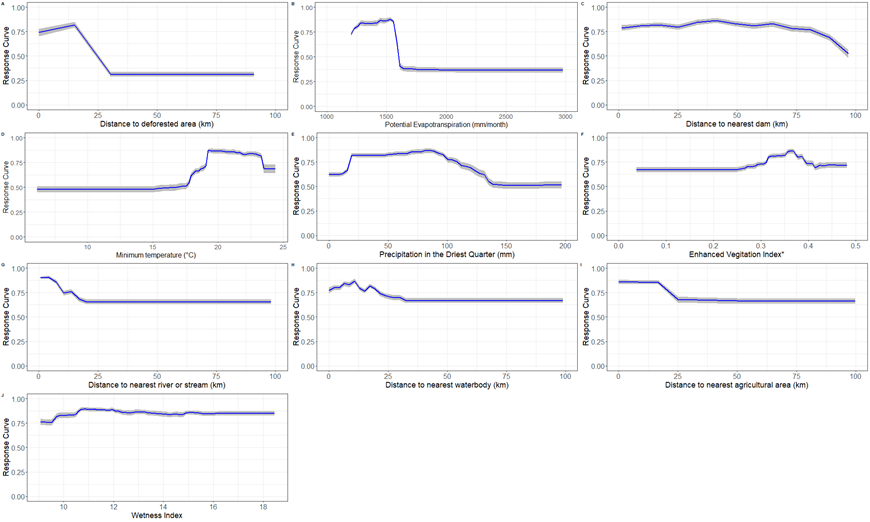

Supplement: S15 Fig — Marginal Effect of Environmental Predictors on Environmental Suitability for Mycobacterium ulcerans. Variables are plotted in order of their contribution to the random forest model. Marginal effect plots illustrate the effect of each explanatory variable on the outcome of suitability for Buruli ulcer. Variables are plotted in order of their contribution to the random forest model. *Interpretation of Enhanced Vegetation Index: low values (0.1–0.15) represent areas of barren rock or sand and built-up land; moderate values (0.15–0.3.5) may indicate shrubs, grassland or cropland; higher values (0.35–0.6) may indicate mixed wood and shrubs or open forest. (TIF) [file pntd.0009157.s018.tif]
